# Dexamethasone’s Clinical Efficacy in Experimental Autoimmune Pancreatitis Correlates with a Unique Transcriptomic Signature, Whilst Kinase Inhibitors Are Not Effective

**DOI:** 10.3390/biomedicines12112480

**Published:** 2024-10-29

**Authors:** Ottavia Agrifoglio, Anika Kasprick, Natalie Gross, Marc Wahlig, Emilia Kauffold, Aline Woitas, Artem Vorobyev, Luise Ehlers, Ralf J. Ludwig, Katja Bieber, Robert Jaster

**Affiliations:** 1 Department of Medicine II, Division of Gastroenterology and Endocrinology, Rostock University Medical Center, 18057 Rostock, Germany; 2 Lübeck Institute of Experimental Dermatology and Center for Research on Inflammation of the Skin, University of Lübeck, 23562 Lübeck, Germany; 3 Department of Dermatology, University of Lübeck, 23562 Lübeck, Germany

**Keywords:** autoimmune pancreatitis, MRL/Mp mouse, dexamethasone, tofacitinib, takinib, transcriptome

## Abstract

(1) Background: Autoimmune pancreatitis (AIP) is mainly treated with steroids. Using an AIP mouse model, we investigated two potential alternatives, the transforming growth factor-β-activated kinase 1 inhibitor, takinib, and the Janus kinase inhibitor, tofacitinib. (2) Methods: In a multicenter preclinical study, MRL/MpJ mice were injected with polyinosinic/polycytidylic acid (poly I:C) for two weeks to induce AIP. They were then treated for four weeks with either takinib (25, 50, or 75 mg/kg body weight), tofacitinib (5, 10 or 15 mg/kg), dexamethasone (1 mg/kg), or solvent, while the poly I:C injections were continued. The severity of AIP was assessed histopathologically. Flow cytometry was used to examine lymphocyte subtypes in the spleen and mesenteric lymph nodes. The pancreatic gene expression profiles were analyzed by RNA sequencing. (3) Results: Poly I:C-treated mice developed severe AIP with inflammation, destruction of acinar tissue, and fibrosis. Dexamethasone significantly attenuated the disease, while takinib or tofacitinib had no effects. Dexamethasone also antagonized the effects of poly I:C on the relative frequencies of the AIP-associated lymphocyte subtypes CD4/CD69, CD8/CD44^high^, and CD4/CD25/FoxP3 in the spleen. In the principal component analysis of pancreatic transcriptomics, poly I:C-injected mice treated with tofacitinib, takinib, or solvent clustered together, while untreated and dexamethasone-treated mice formed separate, unique clusters. (4) Conclusions: Dexamethasone effectively reduced AIP severity, while takinib and tofacitinib were ineffective. The unique gene expression profile in dexamethasone-treated mice may provide a basis for identifying new drug targets for AIP treatment.

## 1. Introduction

Although autoimmunity is a rare cause of chronic pancreatitis (CP), it has attracted much attention in recent years. Two reasons for this are the fact that autoimmune pancreatitis (AIP) is an important differential diagnosis for pancreatic cancer and that the treatment of the disease is fundamentally different from other forms of CP. There are two established subtypes of AIP: AIP-1 (lymphoplasmacytic sclerosing pancreatitis) is considered a pancreatic manifestation of IgG4-related disease, while AIP-2 (idiopathic duct-centric pancreatitis) is often associated with inflammatory bowel disease. Regardless of the subtype, AIP usually responds well to corticosteroid treatment [1,2]. However, problems remain, such as the risk of relapse after completion of treatment and the worrying side effects of long-term systemic steroid treatment.

One of the most commonly used animal models for AIP is the MRL/MpJ strain. Older MRL/MpJ mice (≥6 months), especially females, often spontaneously develop histologic changes reminiscent of human AIP, exemplified by lymphoplasmacytic infiltrates, destruction of exocrine tissue, and fibrosis [3,4]. The disease can be induced, accelerated, and aggravated by injections of the synthetic nucleic acid polyinosinic/polycytidylic acid (poly I:C), even in young animals [5]. MRL/MpJ mice treated with poly I:C respond to steroids with a marked improvement in pancreatic histopathology and are, therefore, a potentially valuable tool for investigating new treatment options for AIP [6]. Using this model, we have already identified cyclosporin A and rapamycin as new drug candidates for the treatment of AIP [6]. In further studies, we also took advantage of the MRL/MpJ strain to identify quantitative trait loci (QTLs) of murine AIP, which ultimately led to the discovery of two new risk genes for AIP in mice [7,8]. The first gene is *Map3k7* (mitogen-activated protein kinase kinase kinase 7); this is also known as transforming growth factor-beta-activated kinase 1 (*Tak1*). The MAP3K7 protein acts upstream of important inflammatory regulators such as MAP kinases and NF-ĸB [9,10]. *Map3k7* gene mutations are associated with inflammatory syndromes, including experimental autoimmune myocarditis [11], encephalomyelitis [12], and type 1 diabetes [13]. With takinib, there is a specific inhibitor for MAP3K7/TAK1 available [14,15]. The second gene, *Bach2*, encodes a transcriptional repressor that controls the expression of a cluster of genes with essential functions in T-cell activation, including many cytokines and cytokine receptors, via stretch or super-enhancers in T-cells [16]. *BACH2* haploinsufficiency can lead to a syndrome of *BACH2*-associated immunodeficiency and autoimmunity [17]. Furthermore, polymorphisms in the human gene locus are known to be associated with multiple autoimmune diseases, including type 1 diabetes [18], Crohn’s disease [19], celiac disease [20], and multiple sclerosis [21]. Transcriptional repression of the *BACH2* gene in CD4^+^ T-lymphocytes of CP patients has been suggested as a key event that drives polarization of pathogenic Th17-cells. To date, there are no drugs known to directly enhance or restore BACH2 function. However, studies have shown that key target genes of the BACH2 protein are inhibited by the Janus kinase (JAK) 1/3 inhibitor tofacitinib [16].

Here, we investigated the effects of takinib and tofacitinib in poly I:C-treated MRL/MpJ mice. Unlike the corticosteroid dexamethasone, neither drug led to an improvement in pancreatic histopathology. Although TAK1 and JAK remain potential targets, their inhibition alone is not sufficient to improve the course of experimental AIP in mice.

## 2. Materials and Methods

### 2.1. Animals

For this multicenter preclinical trial, MRL/MpJ mice were purchased from The Jackson Laboratory (Bar Harbor, ME, via Charles River Laboratories, Sulzfeld, Germany) and maintained as independent mouse colonies in the local animal facilities of the Rostock University Medical Center and the University Hospital Schleswig-Holstein in Lübeck. In pilot experiments, we found that poly I:C-triggered AIP affects both sexes equally.Therefore, and because of the higher clinical relevance, female and male mice were used equally in this study. Mice were held under specific pathogen-free conditions at a 12 h light/dark cycle. They had access to water and rodent chow diet ad libitum. Animal experiments were conducted according to the European Community rules for animal care, approved by the respective governmental administrations (Landesamt für Landwirtschaft, Lebensmittelsicherheit und Fischerei Mecklenburg-Vorpommern and Ministry for Energy, Agriculture, the Environment and Rural Areas Schleswig-Holstein, respectively) and performed by certified personnel.

### 2.2. Induction of AIP and Treatment of MRL/MpJ Mice

**Figure 1:**
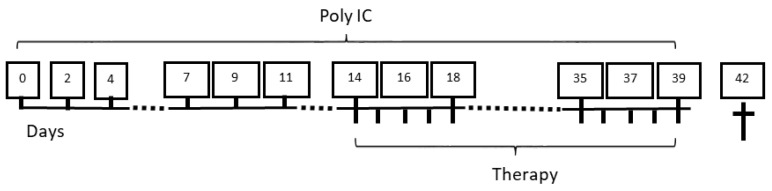
Treatment plan for MRL/MpJ mice. Poly I:C was injected three times per week over a period of 6 weeks. Therapies were administered 5 consecutive days per week for the last 4 weeks and included injections of dexamethasone or administration of takinib and tofacitinib by oral gavage. Please refer to the text in the Section 2 for details.

Mice aged 3 to 4 months were randomly divided into the experimental groups with equal sex distribution. They received intraperitoneal injections of poly I:C (5 µg/g body weight; Miltenyi Biotec, Bergisch Gladbach, Germany; solvent: NaCl 0.9%) for 6 weeks. The specific treatments were initiated after 2 weeks of poly-I:C application and continued until the end of week 6. Takinib and tofacitinib were dissolved in methylcellulose (0.5%) and administered by oral gavage at three different concentrations: Takinib (Hölzel Diagnostika, Cologne, Germany) at a dosage of 25, 50, or 75 mg/kg (designated Tak_L, Tak_M, and Tak_H), and tofacitinib (BIOZOL Diagnostica, Eching, Germany) at a dosage of 5, 10, or 15 mg/kg (designated Tof_L, Tof_M, and Tof_H). Another group of mice was treated with intraperitoneal injections of dexamethasone (Dex) at 1 mg/kg (Merck, Darmstadt, Germany) (solvent: phosphate-buffered saline pH 7.4). Mice with poly I:C injections, but without specific treatment, served as controls (referred to as control+poly I:C). These mice received methylcellulose (0.5%) by oral gavage at the same intervals as the Tak/Tof groups. A second control group neither received poly I:C injections nor any treatment (referred to as control–poly I:C). Each of these groups consisted of 8 female and 8 male mice. In the multicenter setting, exactly half of the experiments were performed at both study sites. The precise treatment protocol is depicted in Figure 1.

On day 42, all mice were sacrificed by an overdose of ketamine/xylazine hydrochloride, followed by cervical dislocation. Blood samples, pancreas, spleen, mesenteric lymph nodes, and further tissues were harvested and stored under appropriate conditions (as detailed below) until they were assayed.

### 2.3. Histology and Immunohistochemistry

Paraffin-embedded pancreatic sections (4 µm thick) were stained with hematoxylin and eosin (H&E). The severity of AIP was assessed by scoring the pancreatic lesions on a semi-quantitative scale from 0 to 4, as previously described [3,6]. Stage 0 is characterized by the absence of any pathological finding. Stage 1 is assigned to pancreata without parenchymal destruction but with minimal focal infiltration of the periductal tissue with mononuclear cells, and stage 2 is assigned to samples with larger periductal foci of mononuclear cells and minimal parenchymal destruction. Severe and multifocal periductal inflammation, together with more extensive parenchymal destruction, are the characteristics of stage 3. Samples with massive infiltration of the pancreatic tissue with mononuclear cells, extensive destruction of the acini, and displacement by adipose or fibrotic tissue are classified as stage 4. All samples were evaluated in a blinded fashion by two independent investigators who assessed at least three tissue sections per sample by light microscopy.

For further cellular and molecular characterization, deparaffinated pancreatic sections (4 µm thick) were stained with antibodies against CD3 (BIOZOL Diagnostica; #DIA-303) or CD138 (BioLegend, San Diego, CA, USA; #142502) using the ImmPRESS-alkaline phosphatase detection system according to the instructions of the manufacturer (Vector Laboratories, Burlingame, CA, USA). After counterstaining with Mayer’s hemalum solution, the slides were dehydrated by two short incubations in ethanol and xylene each and embedded in Pertex (MEDITE, Burgdorf, Germany). Afterwards, representative images of the stains were taken at 100× magnification. The positive cells in these images were then counted and related to the area of the tissue using QuPath version 0.4.3 [22].

### 2.4. Flow Cytometry

Single-cell suspensions from spleens and mesenteric lymph nodes were obtained by passing them through a cell strainer (70 µm) in 10 mL of RPMI1640 medium (PAN Biotech, Aidenbach, Germany) supplemented with fetal calf serum (FBS SUPERIOR stabil, Bio&Sell, Feucht, Germany), penicillin/streptomycin (10,000 U/mL, Thermo Fisher Scientific, Waltham, MA, USA), and 50 µg/mL DNAse I (Roche, Darmstadt, Germany). Red blood cells were lysed by applying 4 mL of ice-cold ammonium chloride (0.25 M in distilled water). After washing steps, the isolated cells were resuspended in a freezing medium (10% dimethyl sulfoxide in fetal calf serum) and stored in liquid nitrogen until further use. After thawing, 1 × 10^6^ viable cells per sample were stained with a Zombie NIR™ Fixable Viability Kit (BioLegend) for 15 min at room temperature (RT) in PBS followed by a 10 min incubation at 4 °C with True-Stain Monocyte Blocker (BioLegend) and FcR Blocking reagent (Miltenyi Biotec). Afterwards, the cfollowing fluorochrome-conjugated antibodies were added to the cells for 20 min at 4 °C: anti-CD4- Brilliant Violet 510 (BioLegend), anti-CD44-PE-Vio770 (Miltenyi Biotec), anti-CD45-PerCP (Miltenyi Biotec), anti-CD69-APC (Miltenyi Biotec), anti-CD8a-Vio Bright FITC (Miltenyi Biotec), anti-CD3e-Brilliant Violet 605 (BioLegend), and anti-CD25-PE (BioLegend). Intracellular staining was performed upon incubation with True-Nuclear Perm Buffer (BioLegend). Subsequently, anti-FoxP3 Brilliant Violet 421 (BioLegend) was added, and the cells were incubated for 30 min at RT. After washing with FACS Buffer, 100,000 cells from each sample were analyzed by flow cytometry using the Cytek™Aurora (Cytek Biosciences, Amsterdam, The Netherlands), and data were evaluated using FlowJo Software (v10.8.1, Tree Star Inc., San Carlos, CA, USA). First, gating was performed to exclude debris, doublets, and dead cells. T-lymphocytes were defined as CD3-positive cells gated from living CD45^+^ cells. Next, CD4^+^ and CD8^+^ cells were selected from the T-lymphocytes and further evaluated by gating CD44^high^, CD69^+^, and CD25^+^FoxP3^+^ cells (the latter only of CD4^+^ cells), respectively.

### 2.5. Gene Expression Analysis by RNA Sequencing (RNA-Seq)

Total RNA was isolated from pancreatic tissue using the Tissue Lyser LT and the RNeasy Mini Kit (both from Qiagen, Hilden, Germany) according to the manufacturer’s instructions. Total RNA samples were quantified by spectrophotometry (NanoDrop 1000, Thermo Fisher Scientific), and their integrity was confirmed using the Agilent Bioanalyzer 2100 with an RNA Nano chip kit (both from Agilent Technologies, Waldbronn, Germany). The validated samples were sent to Novogene (Cambridge, UK) for further sample processing. After mRNA library preparation, RNA-seq was performed using the NovaSeq X Plus System (Illumina, Cambridge, UK) and a paired-end 150 bp (PE150) reading strategy. The number of clean reads varied in the range of 1–2 × 10^9^ per sample.

### 2.6. Routine Laboratory Parameters

Activities of lipase and levels of glucose in blood plasma were determined using standard laboratory assays.

### 2.7. Statistics

The sample size for the multicenter preclinical study was calculated based on the following assumptions: The primary endpoint was pancreatic histopathology as assessed by scoring (see Section 2.3). We calculated with an α-error of 0.05 and a power of 85%. Using SigmaPlot 13.0 (Grafiti LLC, Palo Alto, CA, USA), it was calculated that a group size of 16 mice was required to detect a difference of 0.5 units in histologic score (with an expected SD of 0.33).

Evaluation of RNA-seq data, including group comparisons and parts of statistical analyses, was carried out by Novogene. Additionally, the obtained FASTQ files were aligned to the mouse genome (GRCm39) using Hisat2 [23] and sorted using Samtools [24]. The abundance of counts for the known and novel transcripts was summarized using featureCounts [25]. The output of featureCounts was further analyzed using DESeq2 (version 1.44.0) [26]. Differential expression of genes was analyzed using the Wald test, and genes with padj < 0.05 were considered significant. LFC shrinkage was performed using the ashr package (version 2.2-63) [27]. Gene set enrichment analysis of GO pathways in the detected genes was performed using clusterProfiler (version 4.12.0) [28]. Heatmaps were produced using ggplot2 package (version 3.5.1) [29]; for the volcano plots, EnhancedVolcano package was used (version 1.22.0) [30]. The other data were analyzed with GraphPad Prism (GraphPad Software, version 9.4.1, San Diego, CA, USA). All datasets contained non-normally distributed samples and are, therefore, presented as box plots (interquartile range, median, minimum, and maximum). The Kruskal–Wallis test (unpaired samples) and Dunn‘s multiple comparison tests were employed for their statistical evaluation. Adjusted *p*-values of <0.05 were considered statistically significant.

## 3. Results

### 3.1. Effects of Dexamethasone, Tofacitinib, and Takinib on AIP Severity

**Figure 2:**
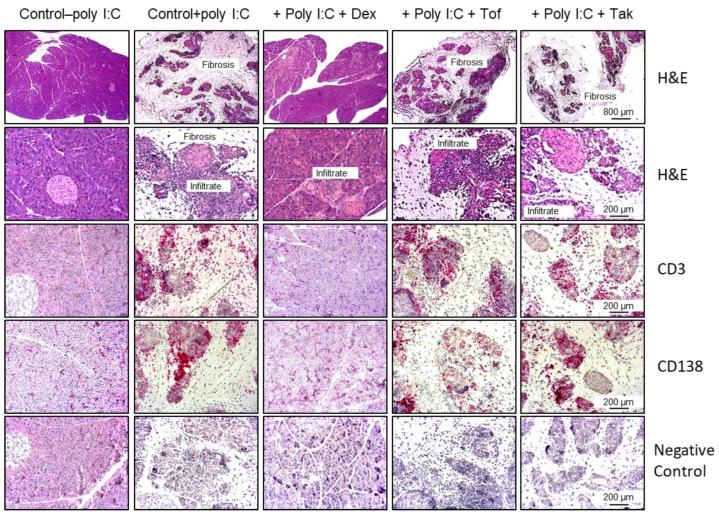
H&E staining of pancreatic tissue and immunohistochemical analysis of inflammatory cell infiltrates. The mice (16 animals per group, equal sex distribution) were either left untreated (control–poly I:C), received injections of poly I:C but treatment with solvent only (control+poly I:C), or were additionally treated with dexamethasone (Dex, 1 mg/kg), tofacitinib (Tof, 15 mg/kg), and takinib (Tak, 75 mg/kg), as indicated. H&E staining: The photographs (original magnification—upper row: 25×; lower row: 100×) display representative examples of pancreatic lesions for each group, scored as stage 0 (healthy pancreas; group control–poly I:C), stage 2 (moderate focal lymphocytic infiltration and locally restricted destruction of acinar tissue; group poly I:C+Dex), and stage 4 (extensive destruction of acinar tissue, severe inflammation, and fibrosis; all other groups). Immunohistochemical analyses: The samples were subjected to immunostaining of CD3 and CD138 as indicated. The negative controls were only treated with the secondary antibody (which was identical for CD3 and CD138 staining). Positively stained cells appear red/magenta. Representative microscopic images (original magnification: 100×) are shown for each group.

**Figure 3:**
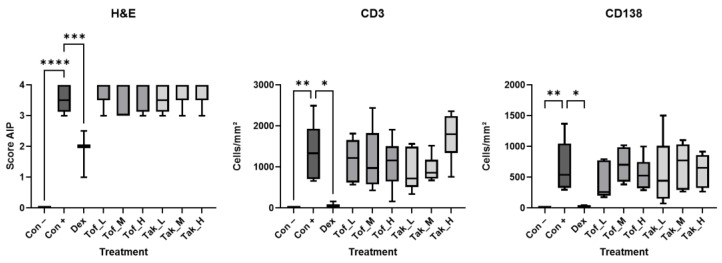
Evaluation of pancreatic histopathology. The mice were either left untreated (control–poly I:C; Con −), received injections of poly I:C but treatment with solvent only (control+poly I:C; Con +), or were additionally treated with dexamethasone (Dex, 1 mg/kg), tofacitinib at 5, 10, or 15 mg/kg (Tof_L, Tof_M, and Tof_H), or takinib at 25, 50, or 75 mg/kg (Tak_L, Tak_M, and Tak_H) as indicated. Left panel: Serial sections of pancreatic tissue were stained with H&E, and lesions were evaluated by applying a scoring system from 0 to 4, as further described in the methods section (16 mice per experimental group, equal sex distribution). Middle and right panels: Tissue sections of 3 male and 3 female mice per group were stained using primary antibodies against CD3 and CD138, as indicated, and the positively stained cells were quantified. Data are expressed as box plots (interquartile range, median, minimum, and maximum). * *p* < 0.05, ** *p* < 0.01, *** *p* < 0.001, **** *p* < 0.0001 versus controls+poly I:C (Kruskal–Wallis test, post hoc analysis: Dunn‘s multiple comparison test).

Completely untreated female and male MRL/MpJ mice aged 3–4 months showed no signs of pancreatic inflammation, as indicated by the H&E staining results (Figure 2). This was accompanied by the absence of CD3^+^ T-cells and CD138^+^ plasma cells (Figure 2 and Figure 3). By contrast, poly I:C administration induced severe pancreatic inflammation with immune cell infiltrates throughout the pancreas and replacement of acinar cells with fibrotic tissue (Figure 2), resulting in an AIP score of 3–4 (Figure 3). The pancreatic infiltrates of poly I:C-exposed mice contained CD3^+^ T-cells and CD138^+^ plasma cells in large numbers, suggesting CP with autoimmune features (Figure 2 and Figure 3).

Consistent with the findings of our previous studies [6], dexamethasone at a dose of 1 mg/kg improved pancreatic inflammation, as evidenced by the absence of extensive immune cell infiltrates, preservation of exocrine pancreatic tissue, and absence of fibrosis (Figure 2). Accordingly, a significantly improved AIP score and a greatly reduced number of CD3^+^ T-cells and CD138^+^ plasma cells were observed (Figure 2 and Figure 3). In contrast, four weeks of treatment with tofacitinib or takinib, each at three different doses, had no significant effect on inflammation and fibrosis and resulted in an unchanged AIP score compared to poly I:C injection alone (Figure 2 and Figure 3).

**Figure 4:**
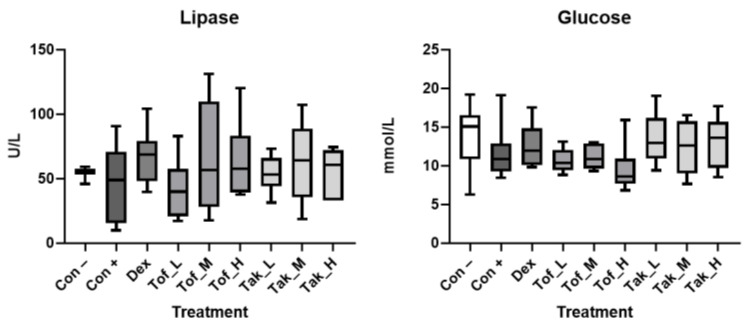
Clinical chemistry. Plasma samples of treated and control mice (as specified in Figure 3) were subjected to the determination of lipase activities and glucose levels. Data from 8 mice per experimental group (with equal sex distribution) are expressed as box plots (interquartile range, median, minimum, and maximum). The Kruskal–Wallis test did not reveal statistically significant differences (*p* < 0.05) between the experimental groups.

In all mice, the pancreatic islets remained largely intact, and in line with this, the plasma glucose levels did not differ between the experimental groups. The chronic pancreatic damage was also not accompanied by increased lipase activities (Figure 4).

### 3.2. Analysis of AIP-Associated Lymphocytic Subpopulations

**Figure 5:**
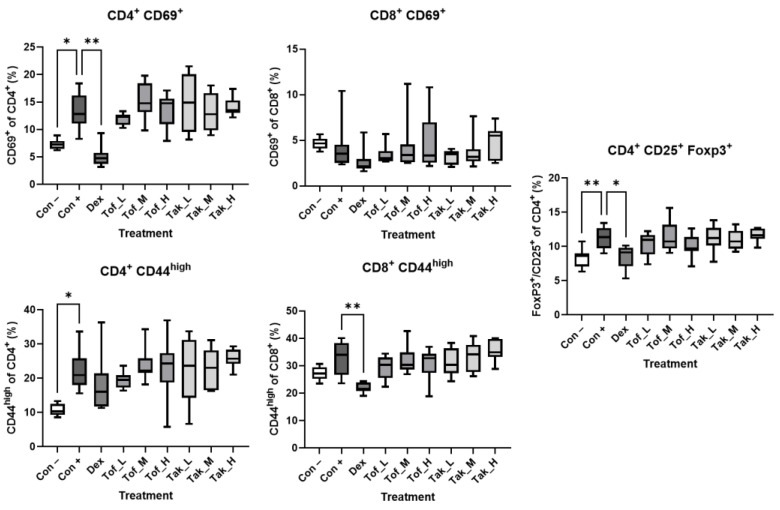
Relative frequencies of AIP-associated lymphocyte subtypes in the spleen. Cells isolated from the spleens of treated and control mice of both sexes (as specified in Figure 3; n = 7–8 per group) were subjected to flow cytometry using labeled antibodies against CD antigens and the intranuclear protein FoxP3, and the relative frequencies of the indicated lymphocyte subpopulations were determined. Data are presented as box plots (interquartile range, median, minimum, and maximum). * *p* < 0.05, ** *p* < 0.01 versus control+poly I:C (Kruskal–Wallis test, post hoc analysis: Dunn‘s multiple comparison test).

**Figure 6:**
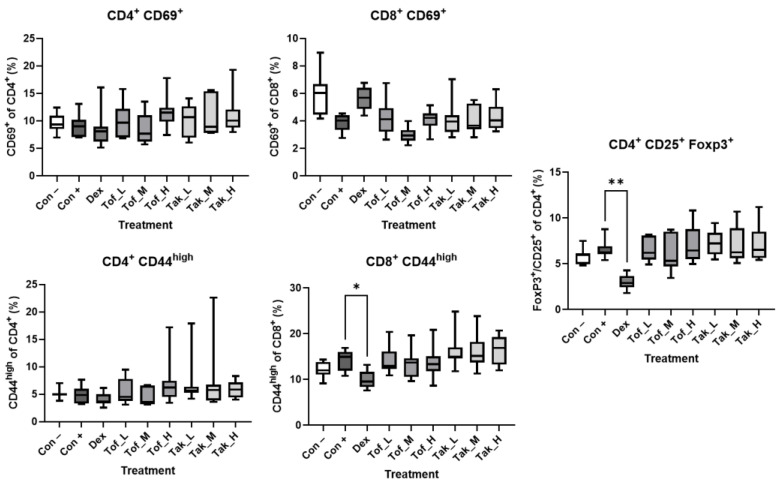
Relative frequencies of AIP-associated lymphocyte subtypes in mesenteric lymph nodes. Cells isolated from the mesenteric lymph nodes of treated and control mice of both sexes (as specified in Figure 3; n = 7–8 per group) were subjected to flow cytometry using labeled antibodies against CD antigens and the intranuclear protein FoxP3, and the relative frequencies of the indicated lymphocyte subpopulations were determined. Data are presented as box plots (interquartile range, median, minimum, and maximum). * *p* < 0.05, ** *p* < 0.01 versus control+poly I:C (Kruskal–Wallis test, post hoc analysis: Dunn‘s multiple comparison test).

We previously identified a quantitative trait locus (QTL) for AIP on mouse chromosome 2 that overlapped with QTLs for CD4^+^/CD44^high^ and CD8^+^/CD44^high^ memory T-cells, FoxP3^+^/CD4^+^ and FoxP3^+^/CD8^+^ regulatory T-cells, and CD8^+^/CD69^+^ cytotoxic T-cells. The relative frequency of CD4^+^/CD44^high^ cells in the spleen also correlated positively with the severity of the disease [7,31]. Using a similar marker panel, we now found that induction of AIP by poly I:C injections significantly increased the relative frequencies of CD4^+^/CD69^+^, CD4^+^/CD44^high^, and CD4^+^/CD25^+^/FoxP3^+^ cells in the spleen. The latter two effects were reversed by treating the mice with dexamethasone, which also significantly reduced the relative abundance of CD8^+^/CD44^high^ cells. In contrast, tofacitinib and takinib had no effects (Figure 5). In the mesenteric lymph nodes, treatment with dexamethasone also led to a significant decrease in the proportion of CD8^+^/CD44^high^ and CD4^+^/CD25^+^/FoxP3^+^ cells in the population examined (Figure 6).

### 3.3. RNA-Seq Analyses of Pancreatic Tissue

**Figure 7:**
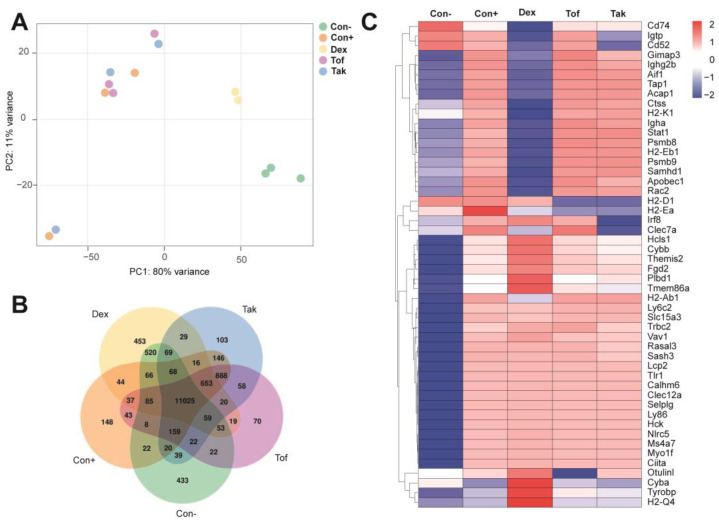
RNA-seq analyses of pancreatic samples. The mice were either left untreated (control–poly I:C; Con −), received injections of poly I:C but treatment with solvent only (control+poly I:C; Con +), or were additionally treated with dexamethasone (Dex, 1 mg/kg), tofacitinib (Tof, 15 mg/kg) and takinib (Tak, 75 mg/kg). The diagrams are based on the analysis of 3 male mice (Dex: 2 mice) per experimental group. (**A**) Principal component analysis (PCA) of gene expression profiles; (**B**) coexpression Venn diagram indicating the number of uniquely expressed genes in each group, with the overlapping regions showing the number of genes that are coexpressed in two or more groups; (**C**) clustering heatmap of the top 50 differentially expressed genes (up- or downregulated) from the comparison Con + versus Con −, based on the adjusted *p*-values. A list of the top 200 genes, along with log2FoldChanges and the corresponding adjusted *p*-values for all comparisons, is provided in Supplementary Materials Table S1. The adjustment for multiple hypothesis testing was performed using the Benjamini–Hochberg false discovery rate method.

To gain insight into the pancreatic gene expression profiles of untreated and treated MRL/MpJ mice, pancreatic tissue was subjected to RNA-seq. Principal component analysis (PCA) revealed that completely untreated mice (control–poly I:C) formed a cluster (Figure 7A) that was distinct from all other groups. The groups of poly I:C-injected mice treated with tofacitinib, takinib, and solvent only (controls+poly I:C) formed a common cluster, while the dexamethasone-treated mice were clustered separately. As shown in the Venn diagram (Figure 7B), the groups of completely untreated mice and mice with poly I:C + dexamethasone had the largest number of uniquely expressed genes (n = 433 and 453, respectively) but also a high number of genes that were coexpressed exclusively in these two groups (n = 520). The other three groups with poly I:C injections (mice treated with tofacitinib or takinib and controls) had only a few uniquely expressed genes (n = 70, 103, and 148, respectively). At the same time, they coexpressed 888 genes (the highest number besides genes expressed in all groups) that were not expressed in completely untreated mice and mice with poly I:C + dexamethasone. A clustering heatmap of differentially expressed genes also showed that the gene expression profile of poly I:C-injected mice with dexamethasone treatment partially overlapped with that of healthy mice without poly I:C injections (Figure 7C). Most of the genes in which dexamethasone attenuated or even reversed the effect of poly I:C are related to immune cell function and inflammation, such as histocompatibility antigens, receptors on immune cells, and mediators of pro-inflammatory signals. Comparison of untreated mice and mice that received injections of poly I:C but were treated with solvent only with mice that were additionally treated with dexamethasone showed that dexamethasone reversed the induction of AIP (Figure S1A). Interestingly, dexamethasone mostly affected several important immune cell pathways in the pancreas (i.e., T-cell activation and adaptive immune response) as well as mitochondrial changes and the ATP synthesis induced by AIP but also changed several GO pathways that were independent (i.e., ribosome biogenesis and gluconeogenesis) of induction of AIP (Figure S1B). Overall, the RNA-seq data are consistent with a therapeutic effect of dexamethasone and confirm the missing therapeutic effects of tofacitinib and takinib.

The top 20 up- and downregulated genes in response to dexamethasone treatment are listed in Table 1 (reference: mice treated with poly I:C only; ranking based on *p* values). All but one gene were found to be downregulated. Most of them are related to the adaptive immune response, which is consistent with the immunosuppressive effect of the drug. The upregulated gene, Klk1b24, encodes for a member of the kallikrein 1-related family of peptidases [24]. To the best of our knowledge, this enzyme has not been linked to pancreatic pathophysiologies before.

## 4. Discussion

The MRL/MpJ mouse strain is a valuable model for studying the pathophysiology of AIP. The disease in this strain mimics many histological features of human AIP-1, including storiform fibrosis, infiltrating IgG4-positive plasma cells, and obliterative phlebitis [3,4,5,6]. A limitation of the model is the absence of IgG4 in mice. The AIP model has previously been used by us and others for the successful testing of experimental therapies, such as the use of rapamycin [6], cyclosporine A [6], and blockade of the interferon-α/interleukin-33 axis [32]. However, all of these approaches still require validation in humans.

By fine-mapping a QTL on mouse chromosome 4, we recently identified *Map3k7* and *Bach2* as susceptibility genes of murine AIP [7]. However, the question of whether the two genes are also suitable as therapeutic targets has not yet been answered. In a straightforward approach, we treated MRL/MpJ mice with poly I:C-induced AIP with the MAP3K7 inhibitor takinib [14] and the JAK inhibitor tofacitinib, which preferentially inhibits the expression of Bach2-regulated pro-inflammatory genes [16]. To achieve reliable results, we endeavored to take a particularly robust approach that improves preclinical animal research’s external validity and reproducibility [33]. The studies were carried out in parallel at two sites with independent mouse colonies and on animals of both sexes. The selected doses covered a range of concentrations previously successfully tested in mouse models [14,34,35]. The application routes also followed established procedures [34,35]. Dexamethasone provided a positive control for treatment effects [6]. Without exception, all mice with poly I:C injections developed the full histologic picture of severe AIP. Consistent with the chronic inflammatory process, the lipase levels were not higher than in untreated control animals. The restriction of the inflammation to the exocrine pancreas was confirmed by the blood glucose concentrations, which also did not deviate from control animals. Dexamethasone showed the expected effect by significantly attenuating inflammation, halting the loss of exocrine pancreatic tissue, and reducing pancreatic fibrosis. The drug also diminished the relative frequencies of lymphocytic subpopulations in the spleen (CD4^+^/CD69^+^, CD8^+^/CD44^high^, and CD4^+^/CD25^+^/FoxP3^+^ cells) with potential roles in murine AIP [6,31]. Finally, the RNA-seq analysis also revealed clear effects of dexamethasone, which were reflected both in the PCA and in a specific decrease in the expression of B- and T-cell-associated genes. In contrast, mice treated with tofacitinib or takinib did not differ in any of the parameters examined from mice injected with poly I:C only. From these data, we conclude that takinib and tofacitinib are ineffective as single agents in the treatment of AIP in mice. One reason for the failure of tofacitinib treatment could be that the targets of the drug only partially overlap with genes downstream of Bach2. Follow-up studies should consider drugs that more accurately reflect the effects of Bach2. The target of takinib, MAP3K7, is, in turn, only one of many potential activators of MAP kinases and NF-ĸB. It is, therefore, conceivable that the loss of MAP3K7 activity is compensated by cytokine receptor-derived pro-inflammatory signals mediated by other members of the MAP kinase kinase kinase family. However, both tofacitinib and takinib could be reconsidered in combination with other drugs that specifically interfere with signaling pathways involved in the development of AIP (see below).

Interestingly, some of the genes regulated by dexamethasone indicate specific effects of the steroid that may go beyond pure immunosuppression. In addition to the aforementioned *Klk1b24*, these include *Ctss, Apobec1*, and *Psmb8*, whose expressions were induced by poly I:C but diminished by dexamethasone. The lysosomal protease cathepsin S (Ctss) has previously been associated with type 1 diabetes [36]. Recently, a specific cathepsin inhibitor, ASPER-29, was found to suppress pancreatic cancer cell metastasis through dual inhibition of cathepsin-L and cathepsin-S [37]. Testing the compound in experimental AIP may be worthwhile. *Apobec1* (apolipoprotein B mRNA editing enzyme, catalytic polypeptide 1) has been identified by bioinformatic analyses as a gene associated with pancreatitis and pancreatic cancer, although a specific role in AIP has not yet been established [38,39]. *Psmb8* (proteasome subunit beta type-8) encodes the catalytic immunoproteasome subunit, has been linked to various cancers, and could be targeted by the selective alpha1a adrenoreceptor antagonist Fiduxosin, according to in silico studies [40]. Thus, there is a unique gene expression profile in the pancreas of dexamethasone-treated mice. It could also provide a basis for identifying new targets for the treatment of AIP.

## Figures and Tables

**Table 1:** Top 20 genes up- and downregulated in dexamethasone-treated mice *.

Gene_Name	Gene_Description	log2Fold Change	*p* (Adjusted)
*Iglc2*	*immunoglobulin lambda constant 2*	−6.75	4.98 × 10^−67^
*Ighg3*	*Immunoglobulin heavy constant gamma 3*	−9.25	2.02 × 10^−61^
*Ly6c2*	*lymphocyte antigen 6 complex*	−6.73	4.44 × 10^−51^
*Igkc*	*immunoglobulin kappa constant*	−7.69	8.92 × 10^−50^
*Lck*	*lymphocyte protein tyrosine kinase*	−3.92	1.53 × 10^−47^
*Jchain*	*immunoglobulin joining chain*	−6.86	9.37 × 10^−47^
*Igkv1-117*	*immunoglobulin kappa variable 1* *–117*	−7.59	1.36 × 10^−40^
*Ltb*	*lymphotoxin B*	−4.75	1.36 × 10^−40^
*Cd3e*	*CD3 antigen*	−4.59	3.13 × 10^−40^
*Cd3d*	*CD3 antigen*	−4.35	1.65 × 10^−37^
*Gimap3*	*GTPase*	−4.37	1.77 × 10^−36^
*Ccr7*	*chemokine (C-C motif) receptor 7*	−4.80	3.78 × 10^−36^
*Cd3g*	*CD3 antigen*	−3.88	5.10 × 10^−36^
*Zap70*	*zeta-chain (TCR) associated protein kinase*	−3.73	6.45 × 10^−36^
*Klk1b24*	*kallikrein 1-related peptidase b24*	11.43	1.81 × 10^−35^
*Cd19*	*CD19 antigen*	−6.16	2.24 × 10^−34^
*Cd2*	*CD2 antigen*	−6.71	9.79 × 10^−33^
*H2-Ob*	*histocompatibility 2*	−5.06	1.51 × 10^−32^
*Rasal3*	*RAS protein activator like 3*	−3.35	8.57 × 10^−32^
*Grap2*	*GRB2-related adaptor protein 2*	−5.05	2.08 × 10^−31^

* The mice either received injections of poly I:C but no specific treatment (n = 3), or were additionally treated with dexamethasone at 1 mg/kg (n = 2). Pancreatic samples were subjected to RNA-seq. A negative change indicates a downregulation in response to the application of dexamethasone.

## Data Availability

Along with the publication of the manuscript, the complete RNA-seq data will be available in the Gene Expression Omnibus database [41] (GEO accession number: GSE277452). Further raw data supporting the conclusions of this article will be made available by the authors upon request.

## Supplementary Materials

The following supporting information can be downloaded at: https://www.mdpi.com/article/10.3390/biomedicines12112480/s1, Figure S1: RNA-seq analyses of pancreatic samples; Table S1: RNA-seq analyses of pancreatic samples.
