# Supplementary material for: Dexamethasone’s Clinical Efficacy in Experimental Autoimmune Pancreatitis Correlates with a Unique Transcriptomic Signature, Whilst Kinase Inhibitors Are Not Effective
Source: Biomedicines. 2024 Oct 29;12(11):2480. doi: 10.3390/biomedicines12112480 (PMC11591683; doi:10.3390/biomedicines12112480)
Supplement: Supplementary file 1 [file biomedicines-12-02480-s001.zip › biomedicines-3250872-supplementary.pdf]

Supplemental data to the manuscript

Dexamethasone's clinical efficacy in experimental autoimmune pancreatitis correlates with a unique transcriptomic signature, whilst kinase inhibitors are not effective

Authors: Ottavia Agrifoglio [1, †], Anika Kasprick [2, †], Natalie Gross [2], Marc Wahlig [1], Emilia Kauffold [1], Aline Woitas [1], Artem Vorobyev [3], Luise Ehlers [1], Ralf J. Ludwig [2, 3], Katja Bieber [2, ‡], and Robert Jaster [1, *,‡]

[1] Department of Medicine II, Division of Gastroenterology and Endocrinology, Rostock University Medical Center, Rostock, Germany

[2] Lübeck Institute of Experimental Dermatology and Center for Research on Inflammation of the Skin, University of Lübeck, Germany

[3] Department of Dermatology, University of Lübeck, Germany

* Correspondence: robert.jaster@med.uni-rostock.de; Tel.: +49-381-4947349

† These authors contributed equally to this work.

‡ These authors contributed equally to this work.

Table S1. RNA-seq analyses of pancreatic samples. The mice were either left untreated (control [C]-poly I:C), received injections of poly I:C but treatment with solvent only (c+poly I:C), or were additionally treated with dexamethasone (Dex, 1 mg/kg), tofacitinib (Tof, 15 mg/kg) or takinib (Tak, 75 mg/kg). Each group consisted of three male mice (Dex: 2 animals). Listed are the top 200 genes (up- or downregulated) from the comparison C+poly:IC versus C-poly:IC, based on the adjusted p-values (calculated using the Benjamini-Hochberg false discovery rate [FDR] method to adjust for multiple hypothesis testing). For each gene, the ID, the official gene symbol, the log2FoldChange for all comparisons (each group against every other group) and the corresponding adjusted p-value (padj) are displayed.

Figure S1. RNA-seq analyses of pancreatic samples. The mice were either left untreated (control-poly I:C; Con -), received injections of poly I:C but treatment with solvent only (control+poly I:C; Con +), or were additionally treated with dexamethasone (Dex, 1 mg/kg), tofacitinib (Tof, 15 mg/kg) and takinib (Tak, 75 mg/kg). Data are from three male mice (Dex: 2 mice) per experimental group. (A): Volcano Plot of the comparison of Con -/ Con + and Con +/ Dex showed a clear reversal of the AIP-regulated genes by Dex. The top 50 genes (from Figure 7B) are indicated in both plots. (B): Heatmap of Gene set enrichment analysis (GSEA) of gene ontology (GO) pathways of the 20 most prominent pathways regulated by Con -/ Con + and Con +/ Dex.

Table S1

Gene_id	Gene_name	C+poly:IC vs C-poly:IC _log2 FoldChange	C+poly:IC vs C -poly:IC _padj	Tof vs C- poly:IC _log2 FoldChange	Tof vs C-poly:IC _padj	Tak vs C- poly:IC _log2 FoldChange	Tak vs C- poly:IC _padj	Dex vs C-poly:IC _log2 FoldChange	Dex vs C-poly:IC _padj	Tak vs Tof _log2 FoldChange	Tak vs Tof _padj	Tof vs Dex _log2 Fold Change	Tof vs Dex _padj	Tak vs Dex_log2 Fold Change	Tak vs Dex _padj	Tof vs C+poly:IC _log2 FoldChange	Tof vs C+ poly:IC _padj	Tak vs C+poly:IC_ log2 FoldChange	Tak vs C+ poly:IC _padj	Dex vs C+poly:IC _log2 FoldChange	Dex vs C+ poly:IC _padj
ENSMUSG00000038642	Ctss	6.E+14	3.27E-77	5.74E+14	6.68E-90	5.96E+14	3.87E-46	3.59E+14	7.16E-27	2.E-01	1.00E+00	2.E+14	4.89E-06	2.E+14	7.56E+05	4.10E-02	1.00E+00	2.18E-01	1.00E+00	-2.08E+14	8.57E+00
ENSMUSG00000041515	Irf8	5.E+14	6.67E-61	4.82E+14	3.84E-62	4.84E+14	1.74E-28	2.03E+14	1.07E+07	-7.E-03	1.00E+00	3.E+14	2.92E-19	3.E+14	3.30E+01	4.14E-02	1.00E+00	2.39E-02	1.00E+00	-2.71E+14	6.09E-15
ENSMUSG00000074151	Nlrc5	5.E+14	1.65E-57	5.40E+14	4.56E-86	5.41E+14	2.13E-77	3.82E+14	1.77E-15	-1.E-02	1.00E+00	2.E+14	1.14E+06	2.E+14	8.47E+08	1.83E-01	1.00E+00	1.59E-01	1.00E+00	-1.36E+14	1.50E-04
ENSMUSG00000037321	Tap1	5.E+14	5.57E-51	4.83E+14	4.06E-70	4.83E+14	1.22E-43	3.24E+14	3.95E-11	-2.E-02	1.00E+00	2.E+14	6.21E+03	2.E+13	1.48E+09	7.72E-02	1.00E+00	4.23E-02	1.00E+00	-1.47E+14	1.93E+08
ENSMUSG00000022584	Ly6c2	9.E+14	4.39E-49	8.02E+14	1.63E-47	8.11E+14	4.01E-30	1.96E+13	3.19E-01	7.E-02	1.00E+00	6.E+14	1.90E-49	6.E+13	9.73E-19	-7.23E-01	1.00E+00	-6.65E-01	1.00E+00	-6.73E+14	4.44E-37
ENSMUSG00000037731	Themis2	5.E+14	1.03E-45	5.12E+14	1.41E-86	5.30E+13	9.86E-40	2.63E+14	4.32E+03	2.E-01	1.00E+00	2.E+14	3.06E-22	3.E+13	5.22E+01	4.53E-02	1.00E+00	1.86E-01	1.00E+00	-2.41E+14	2.22E+00
ENSMUSG00000024737	Slc15a3	5.E+13	1.02E-39	4.76E+14	4.67E-31	4.97E+14	3.42E-28	2.24E+13	7.55E-03	2.E-01	1.00E+00	2.E+14	1.61E+01	3.E+14	1.16E+05	-2.50E-02	1.00E+00	1.52E-01	1.00E+00	-2.51E+14	2.24E+02
ENSMUSG00000030579	Tyrobp	4.E+14	4.15E-38	4.13E+14	8.34E-34	4.34E+14	7.13E-15	2.13E+14	6.93E+05	2.E-01	1.00E+00	2.E+14	3.58E-03	2.E+14	3.58E+07	2.19E-01	1.00E+00	3.95E-01	1.00E+00	-1.74E+14	1.07E-03
ENSMUSG00000024397	Aif1	6.E+14	2.26E-37	5.94E+14	1.59E-40	6.12E+14	1.79E-23	3.09E+13	2.59E+08	2.E-01	1.00E+00	3.E+14	7.97E-16	3.E+14	7.79E+00	1.10E-01	1.00E+00	2.51E-01	1.00E+00	-2.71E+14	4.76E-10
ENSMUSG00000024300	Myo1f	5.E+14	1.14E-36	4.84E+14	2.07E-66	4.98E+14	4.01E-29	2.59E+13	2.26E+04	1.E-01	1.00E+00	2.E+14	1.55E-30	2.E+14	1.75E+03	2.77E-02	1.00E+00	1.26E-01	1.00E+00	-2.19E+14	4.15E+00
ENSMUSG00000024338	Psmb8	4.E+14	4.00E-36	4.39E+14	3.64E-45	4.48E+14	9.79E-23	2.79E+14	1.10E-01	6.E-02	1.00E+00	2.E+14	4.73E-01	2.E+14	3.62E+09	1.74E-01	1.00E+00	2.26E-01	1.00E+00	-1.38E+14	4.74E+06
ENSMUSG00000076613	Ighg2b	1.E+14	5.45E-36	6.90E+13	1.99E+05	9.26E+14	7.10E+03	-2.32E-01	9.83E-01	2.E+14	1.00E+00	7.E+14	5.27E+07	9.E+14	2.08E+04	-4.19E+14	3.02E-01	-1.88E+14	1.00E+00	-1.13E+14	8.64E-07
ENSMUSG00000095079	Igha	7.E+14	6.41E-34	7.47E+14	6.68E-36	7.59E+12	9.32E-22	3.08E+14	3.27E+04	7.E-02	1.00E+00	4.E+14	7.37E+03	4.E+14	1.33E+04	3.14E-02	1.00E+00	9.77E-02	1.00E+00	-4.34E+14	2.48E-01
ENSMUSG00000035929	H2-Q4	4.E+14	9.85E-34	3.85E+14	3.59E-42	3.85E+14	2.08E-18	2.41E+14	5.25E+00	-2.E-02	1.00E+00	1.E+14	5.02E+06	1.E+12	1.02E-03	2.01E-01	1.00E+00	1.66E-01	1.00E+00	-1.20E+14	7.94E+09
ENSMUSG00000040613	Apobec1	5.E+14	7.04E-32	4.45E+14	7.94E-52	4.75E+14	6.02E-21	2.65E+14	2.73E+00	3.E-01	1.00E+00	2.E+14	9.87E-17	2.E+14	2.29E+07	-2.33E-01	1.00E+00	2.15E-02	1.00E+00	-1.99E+14	1.00E+04
ENSMUSG00000078853	Igtp	6.E+14	2.10E-31	6.18E+14	3.54E-68	6.10E+14	1.09E-49	4.72E+14	7.20E-27	-1.E-01	1.00E+00	1.E+14	1.96E+08	1.E+14	1.85E-03	4.02E-01	1.00E+00	2.88E-01	1.00E+00	-1.02E+14	4.61E-02
ENSMUSG00000046031	Calhm6	8.E+14	2.25E-31	7.85E+14	1.22E-39	7.95E+14	8.33E-30	4.90E+14	3.14E+08	7.E-02	1.00E+00	3.E+14	4.80E-16	3.E+14	1.42E-05	2.80E-01	1.00E+00	3.41E-01	1.00E+00	-2.64E+14	1.83E-05
ENSMUSG00000076498	Trbc2	5.E+14	3.04E-31	4.94E+14	1.01E-27	5.21E+14	4.72E-23	1.78E+14	2.21E-01	2.E-01	1.00E+00	3.E+14	2.56E+00	3.E+14	6.84E+03	1.72E-02	1.00E+00	2.45E-01	1.00E+00	-3.10E+14	8.89E+01
ENSMUSG00000034116	Vav1	5.E+14	3.14E-31	4.36E+14	7.88E-47	4.51E+14	5.70E-19	1.65E+14	3.10E-03	1.E-01	1.00E+00	3.E+13	1.67E-16	3.E+14	4.89E+02	-1.40E-01	1.00E+00	-3.84E-02	1.00E+00	-2.82E+14	1.73E-04
ENSMUSG00000096727	Psmb9	4.E+14	3.42E-30	4.42E+14	8.76E-45	4.41E+14	3.57E-23	2.66E+14	1.27E+02	-3.E-02	1.00E+00	2.E+14	3.78E+03	2.E+14	4.06E+09	2.35E-01	1.00E+00	1.95E-01	1.00E+00	-1.49E+14	7.46E+08
ENSMUSG00000024672	Ms4a7	5.E+14	3.93E-30	4.81E+14	6.65E-65	4.90E+14	2.39E-18	2.47E+14	3.40E+06	6.E-02	1.00E+00	2.E+14	2.48E-34	2.E+14	2.91E+06	2.26E-01	1.00E+00	2.76E-01	1.00E+00	-2.08E+14	2.22E+01
ENSMUSG00000033220	Rac2	6.E+14	8.35E-29	5.35E+14	6.29E-49	5.47E+14	9.18E-29	2.43E+14	3.88E+05	8.E-02	1.00E+00	3.E+14	5.69E-11	3.E+14	1.55E+02	-1.48E-01	1.00E+00	-7.77E-02	1.00E+00	-3.04E+14	1.01E+02
ENSMUSG00000022504	Ciita	5.E+14	1.40E-28	4.84E+14	2.19E-42	4.87E+14	6.09E-27	2.60E+14	2.18E+05	-4.E-03	1.00E+00	2.E+13	6.46E-18	2.E+14	5.53E-01	9.84E-02	1.00E+00	8.36E-02	1.00E+00	-2.11E+14	8.57E+00
ENSMUSG00000001588	Acap1	5.E+14	1.86E-27	5.27E+14	4.50E-20	5.53E+14	7.13E-15	1.16E+13	4.92E-01	2.E-01	1.00E+00	4.E+14	3.01E-11	4.E+13	2.78E-03	-9.37E-02	1.00E+00	1.08E-01	1.00E+00	-4.18E+14	5.81E-15
ENSMUSG00000073411	H2-D1	3.E+14	2.91E-27	3.41E+14	1.06E-41	3.42E+14	2.25E-16	2.16E+14	1.81E+02	-2.E-02	1.00E+00	1.E+13	5.27E+07	1.E+14	2.34E-03	1.20E-01	1.00E+00	8.78E-02	1.00E+00	-1.09E+14	3.34E-04
ENSMUSG00000061232	H2-K1	4.E+14	2.16E-26	3.75E+14	9.47E-31	3.74E+14	7.06E-18	2.36E+14	4.17E-03	-4.E-02	1.00E+00	1.E+14	1.18E+06	1.E+14	3.17E-04	1.09E-01	1.00E+00	5.98E-02	1.00E+00	-1.25E+14	5.82E+08
ENSMUSG00000073421	H2-Ab1	6.E+14	2.31E-26	5.57E+14	2.88E-31	5.61E+14	1.65E-17	3.28E+14	7.52E+03	1.E-02	1.00E+00	2.E+14	7.43E-06	2.E+14	4.79E-06	3.81E-02	1.00E+00	3.93E-02	1.00E+00	-2.22E+14	1.55E+02
ENSMUSG00000060586	H2-Eb1	5.E+14	3.27E-26	5.28E+13	1.69E-40	5.32E+14	8.12E-22	2.53E+14	9.61E+01	7.E-03	1.00E+00	3.E+14	1.01E-14	3.E+14	1.85E+03	2.24E-01	1.00E+00	2.22E-01	1.00E+00	-2.49E+13	7.29E+01
ENSMUSG00000022831	Hcls1	5.E+14	3.27E-26	4.53E+14	5.01E-35	4.64E+13	7.16E-14	1.91E+14	8.70E-04	8.E-02	1.00E+00	3.E+14	1.16E-16	3.E+14	1.29E+04	-4.13E-02	1.00E+00	2.65E-02	1.00E+00	-2.63E+14	2.86E-05
ENSMUSG00000079293	Clec7a	5.E+13	5.11E-26	4.91E+14	9.72E-29	5.49E+14	2.21E-11	2.99E+14	2.87E+05	5.E-01	1.00E+00	2.E+14	4.47E+01	2.E+14	2.70E+09	-3.53E-01	1.00E+00	1.78E-01	1.00E+00	-2.25E+14	1.35E+05
ENSMUSG00000039264	Gimap3	5.E+14	8.76E-26	4.41E+14	1.85E-16	4.72E+14	4.11E-12	1.76E-01	9.68E-01	3.E-01	1.00E+00	4.E+14	3.04E-17	4.E+14	3.21E-05	-1.67E-01	1.00E+00	9.50E-02	1.00E+00	-4.37E+14	1.77E-22
ENSMUSG00000021423	Ly86	4.E+14	1.52E-25	4.59E+14	6.85E-58	4.55E+14	4.63E-17	1.81E+14	1.02E-02	-7.E-02	1.00E+00	3.E+14	2.08E-07	3.E+13	3.66E+06	1.66E-01	1.00E+00	8.98E-02	1.00E+00	-2.58E+14	1.08E+04
ENSMUSG00000030214	Plbd1	4.E+14	2.43E-25	4.16E+14	9.12E-47	4.11E+14	9.30E-18	1.40E+14	2.64E-03	-9.E-02	1.00E+00	3.E+14	7.43E-19	3.E+14	2.71E+02	9.61E-02	1.00E+00	4.80E-04	1.00E+00	-2.64E+14	1.02E-02
ENSMUSG00000000682	Cd52	6.E+14	6.90E-25	5.80E+14	8.62E-31	6.01E+14	1.71E-14	2.46E+14	1.83E-03	2.E-01	1.00E+00	3.E+14	1.32E-30	3.E+14	1.67E+01	1.45E-01	1.00E+00	3.03E-01	1.00E+00	-3.16E+14	1.26E-13
ENSMUSG00000006519	Cyba	3.E+13	8.56E-25	2.77E+14	8.28E-12	3.03E+14	1.85E-03	1.23E+14	2.78E+08	2.E-01	1.00E+00	2.E+14	1.64E+05	2.E+14	2.55E+09	1.35E-01	1.00E+00	3.52E-01	1.00E+00	-1.37E+14	4.22E-01
ENSMUSG00000036322	H2-Ea	5.E+14	4.13E-24	5.34E+14	1.55E-29	5.44E+14	2.07E-18	2.72E+14	2.60E+04	7.E-02	1.00E+00	3.E+14	1.35E-16	3.E+14	3.34E+01	1.82E-01	1.00E+00	2.39E-01	1.00E+00	-2.39E+14	1.06E-04
ENSMUSG00000026104	Stat1	4.E+13	4.13E-24	4.72E+14	4.24E-123	4.71E+14	1.58E-36	2.88E+14	2.79E-06	-4.E-02	1.00E+00	2.E+14	8.79E-01	2.E+14	2.01E+09	2.85E-01	1.00E+00	2.36E-01	1.00E+00	-1.52E+14	5.79E-04
ENSMUSG00000048163	Selplg	4.E+14	5.06E-24	4.48E+14	1.38E-33	4.42E+13	3.49E-21	1.65E+14	1.18E-02	-8.E-02	1.00E+00	3.E+14	7.09E-12	3.E+14	1.11E+00	2.56E-02	1.00E+00	-6.62E-02	1.00E+00	-2.77E+14	1.38E-02
ENSMUSG00000024013	Fgd2	3.E+14	7.45E-24	3.42E+14	7.81E-52	3.62E+14	1.22E-12	1.30E+14	5.78E-04	2.E-01	1.00E+00	2.E+14	1.57E-33	2.E+14	5.48E+04	1.04E-01	1.00E+00	2.64E-01	1.00E+00	-1.98E+14	7.69E-02
ENSMUSG00000052142	Rasal3	4.E+14	1.33E-23	4.37E+14	8.68E-19	4.46E+14	2.16E-14	9.39E-01	4.32E-01	6.E-02	1.00E+00	3.E+14	4.34E-13	3.E+14	2.13E-04	4.58E-02	1.00E+00	9.60E-02	1.00E+00	-3.35E+14	8.57E-18
ENSMUSG00000010307	Tmem86a	4.E+14	4.35E-23	4.42E+14	7.35E-33	4.67E+14	1.04E-18	2.30E+14	6.05E+08	2.E-01	1.00E+00	2.E+14	1.91E+00	2.E+14	4.06E+05	-7.32E-02	1.00E+00	1.28E-01	1.00E+00	-2.16E+14	2.28E+05
ENSMUSG00000002699	Lcp2	6.E+14	5.17E-23	5.42E+14	1.51E-41	5.60E+14	2.88E-17	2.43E+14	1.65E-03	1.E-01	1.00E+00	3.E+14	3.75E-39	3.E+14	4.63E+00	-9.73E-02	1.00E+00	3.65E-02	1.00E+00	-3.05E+14	1.46E-02
ENSMUSG00000044827	Tlr1	5.E+14	1.06E-22	5.32E+14	2.83E-31	5.46E+14	7.13E-15	2.31E+14	5.52E-03	1.E-01	1.00E+00	3.E+14	4.09E-28	3.E+14	3.99E+00	-1.57E-01	1.00E+00	-6.67E-02	1.00E+00	-3.14E+14	1.28E-06
ENSMUSG00000053063	Clec12a	6.E+14	1.80E-22	5.88E+14	8.21E-35	6.16E+14	5.38E-19	3.31E+14	2.83E+06	3.E-01	1.00E+00	3.E+14	9.45E-48	3.E+14	7.71E+01	-1.48E-02	1.00E+00	2.25E-01	1.00E+00	-2.55E+14	7.08E+00
ENSMUSG00000003283	Hck	5.E+14	3.17E-22	5.56E+14	2.08E-26	5.59E+14	8.27E-18	2.93E+14	9.21E+09	5.E+09	1.00E+00	3.E+14	2.99E-17	3.E+14	2.37E-01	1.41E-01	1.00E+00	1.30E-01	1.00E+00	-2.45E+14	1.71E-07
ENSMUSG00000027639	Samhd1	4.E+14	3.88E-22	4.52E+14	2.25E-37	4.51E+14	1.66E-24	2.26E+14	8.87E+04	-4.E-02	1.00E+00	2.E+14	3.35E-13	2.E+14	1.66E+01	2.08E-01	1.00E+00	1.56E-01	1.00E+00	-2.02E+14	1.83E+04
ENSMUSG00000031101	Sash3	5.E+12	5.56E-22	5.09E+14	1.61E-28	5.12E+14	1.09E-14	1.33E+14	4.03E-01	2.E-03	1.00E+00	4.E+14	3.19E-07	4.E+14	9.11E+02	-4.17E-02	1.00E+00	-5.06E-02	1.00E+00	-3.77E+14	2.08E-01
ENSMUSG00000056069	Otulinl	4.E+14	7.15E-22	3.90E+14	4.38E-41	3.92E+14	6.86E-15	1.71E+14	3.66E-04	-6.E-03	1.00E+00	2.E+14	1.02E-28	2.E+14	1.61E+04	1.74E-01	1.00E+00	1.57E-01	1.00E+00	-1.97E+14	4.35E-02
ENSMUSG00000024610	Cd74	5.E+14	2.66E-21	5.03E+14	8.84E-31	5.11E+13	1.98E-19	2.43E+14	1.43E+03	5.E-02	1.00E+00	3.E+14	5.08E-09	3.E+14	9.54E+02	1.24E-01	1.00E+00	1.61E-01	1.00E+00	-2.44E+14	1.20E+03
ENSMUSG00000015340	Cybb	5.E+14	4.99E-21	4.89E+14	3.58E-38	5.07E+14	2.50E-15	2.49E+13	1.16E+05	2.E-01	1.00E+00	2.E+14	1.46E-16	3.E+14	2.23E+06	-9.22E-02	1.00E+00	4.80E-02	1.00E+00	-2.46E+14	1.80E+04
ENSMUSG00000035004	Igsf6	4.E+14	3.57E-20	3.98E+14	4.31E-22	4.31E+14	5.15E-13	2.18E+14	6.71E-04	3.E-01	1.00E+00	2.E+14	1.65E-02	2.E+14	1.98E+05	-3.67E-02	1.00E+00	2.50E-01	1.00E+00	-1.80E+14	7.75E+00
ENSMUSG00000021880	Rnase6	7.E+14	6.27E-20	7.00E+14	3.33E-30	6.99E+14	2.74E-15	3.89E+14	1.77E-02	-7.E-02	1.00E+00	3.E+14	5.44E-30	3.E+14	4.20E-03	6.49E-02	1.00E+00	-1.75E-02	1.00E+00	-3.08E+14	8.48E-07
ENSMUSG00000040552	C3ar1	5.E+14	1.59E-19	4.82E+14	5.54E-52	5.11E+14	2.48E-30	2.85E+14	1.02E+05	3.E-01	1.00E+00	2.E+14	4.90E-10	2.E+13	8.58E+03	-1.50E-01	1.00E+00	9.72E-02	1.00E+00	-2.09E+14	1.02E+07
ENSMUSG00000046805	Mpeg1	5.E+14	1.63E-19	5.21E+14	3.78E-30	5.20E+14	1.85E-17	2.92E+14	1.56E+02	-4.E-02	1.00E+00	2.E+14	5.90E-11	2.E+14	1.11E+04	-8.54E-03	1.00E+00	-5.67E-02	1.00E+00	-2.26E+13	2.58E+05
ENSMUSG00000024679	Ms4a6d	4.E+14	2.05E-19	4.43E+14	5.47E-29	4.52E+14	5.88E-20	2.54E+14	4.67E+07	7.E-02	1.00E+00	2.E+14	1.55E-03	2.E+13	1.28E+04	2.14E-01	1.00E+00	2.73E-01	1.00E+00	-1.63E+14	2.47E+06
ENSMUSG00000112023	Lilrb4b	4.E+14	2.66E-19	3.92E+14	3.56E-08	4.47E+14	1.19E-11	3.08E+14	7.95E-01	5.E-01	1.00E+00	8.E-01	2.11E-02	1.E+13	1.02E-03	-3.42E-01	1.00E+00	1.64E-01	1.00E+00	-1.15E+12	8.23E+08
ENSMUSG00000036594	H2-Aa	5.E+12	7.14E-19	4.98E+13	4.63E-30	5.05E+14	1.64E-17	2.51E+13	3.40E+03	4.E-02	1.00E+00	2.E+14	7.90E-12	2.E+14	4.30E+03	1.57E-01	1.00E+00	1.90E-01	1.00E+00	-2.27E+14	1.36E+04
ENSMUSG00000047180	Neurl3	4.E+14	8.81E-18	4.30E+14	1.72E-28	4.23E+14	9.41E-08	1.84E+14	1.87E-03	-1.E-01	1.00E+00	2.E+14	6.20E-15	2.E+14	1.72E+07	3.68E-02	1.00E+00	-8.70E-02	1.00E+00	-2.39E+13	1.54E+01
ENSMUSG00000075010	AW112010	4.E+14	9.80E-18	4.35E+14	9.21E-71	4.44E+14	4.72E-23	1.79E+14	2.40E+07	6.E-02	1.00E+00	3.E+14	5.24E-38	3.E+14	2.55E+02	3.37E-01	1.00E+00	3.88E-01	1.00E+00	-2.19E+14	1.11E+05
ENSMUSG00000036908	Unc93b1	4.E+14	1.39E-17	3.81E+14	1.15E-27	3.96E+13	3.70E-12	1.74E+13	4.82E+07	1.E-01	1.00E+00	2.E+14	7.18E-15	2.E+14	1.22E+05	-3.01E-03	1.00E+00	1.15E-01	1.00E+00	-2.03E+14	9.40E+01
ENSMUSG00000031304	Il2rg	4.E+14	1.43E-17	4.27E+14	5.36E-44	4.38E+14	1.02E-11	1.69E+13	6.51E-03	6.E-02	1.00E+00	3.E+14	4.59E-21	3.E+14	2.82E+05	8.64E-02	1.00E+00	1.38E-01	1.00E+00	-2.47E+14	3.89E+02
ENSMUSG00000020077	Srgn	3.E+14	1.88E-17	3.02E+14	4.21E-14	3.25E+14	6.15E+00	1.08E+13	9.03E-03	2.E-01	1.00E+00	2.E+14	1.02E+03	2.E+14	5.44E+09	-1.42E-01	1.00E+00	3.61E-02	1.00E+00	-2.05E+14	4.93E+02
ENSMUSG00000026288	Inpp5d	4.E+14	7.11E-17	4.23E+14	9.89E-36	4.26E+14	2.63E-18	1.88E+13	1.40E-04	4.E-03	1.00E+00	2.E+14	1.07E-18	2.E+14	4.46E+01	-8.61E-02	1.00E+00	-9.36E-02	1.00E+00	-2.40E+14	2.35E+03
ENSMUSG00000052776	Oas1a	5.E+14	7.17E-17	6.14E+14	8.57E-23	5.67E+14	1.36E-17	3.21E+14	1.08E-04	-5.E-01	1.00E+00	3.E+14	1.12E+01	2.E+14	1.43E+06	6.37E-01	1.00E+00	1.31E-01	1.00E+00	-2.26E+14	1.02E+07
ENSMUSG00000011008	Mcoln2	6.E+14	7.75E-17	5.36E+14	2.44E-17	6.03E+14	1.39E-05	3.27E+13	3.76E-03	6.E-01	1.00E+00	2.E+14	4.18E+05	3.E+14	1.55E-04	-5.48E-01	1.00E+00	7.07E-02	1.00E+00	-2.61E+14	2.62E+06
ENSMUSG00000049625	Tifab	5.E+13	8.25E-17	4.78E+14	1.31E-20	5.00E+13	4.89E-15	2.49E+14	3.21E-03	2.E-01	1.00E+00	2.E+14	1.62E+00	2.E+14	6.52E+04	1.54E-01	1.00E+00	3.30E-01	1.00E+00	-2.11E+14	2.23E+04
ENSMUSG00000030149	Klrk1	9.E+14	1.26E-16	9.17E+14	3.53E-21	9.11E+14	1.16E-17	5.82E+14	2.88E+09	-8.E-02	1.00E+00	3.E+14	3.88E-16	3.E+14	2.76E-03	1.84E-01	1.00E+00	9.49E-02	1.00E+00	-3.12E+14	1.46E-01
ENSMUSG00000004266	Ptpn6	3.E+14	1.41E-16	3.31E+14	9.87E-42	3.40E+14	8.76E-07	1.10E+14	1.36E-03	5.E-02	1.00E+00	2.E+14	3.99E-26	2.E+14	1.86E+05	9.56E-02	1.00E+00	1.37E-01	1.00E+00	-2.09E+14	7.08E+00
ENSMUSG00000024339	Tap2	3.E+14	1.95E-16	3.00E+14	3.05E-21	3.03E+14	9.18E-12	1.81E+14	6.35E+07	1.E-02	1.00E+00	1.E+14	3.86E+09	1.E+14	4.28E-03	1.56E-01	1.00E+00	1.58E-01	1.00E+00	-1.00E+14	2.72E-03
ENSMUSG00000024349	Sting1	3.E+14	1.95E-16	3.33E+14	1.24E-21	3.52E+14	7.32E-09	1.29E+14	1.65E-02	2.E-01	1.00E+00	2.E+14	3.71E-05	2.E+14	5.35E+05	-7.87E-02	1.00E+00	6.99E-02	1.00E+00	-2.09E+14	1.06E+01

Table S1

ENSMUSG00000015947	Fcgr1	5.E+14	2.34E-16	4.88E+14	7.28E-29	4.98E+14	6.26E-11	2.22E+14	2.17E-03	7.E-02	1.00E+00	3.E+14	1.25E-14	3.E+13	3.96E+05	1.68E-01	1.00E+00	2.32E-01	1.00E+00	-2.46E+14	1.97E+01
ENSMUSG00000031389	Arhgap4	4.E+14	3.11E-16	3.50E+13	3.25E-22	3.77E+14	1.00E-07	5.38E-01	6.85E-01	2.E-01	1.00E+00	3.E+14	8.34E-11	3.E+14	1.55E+02	-4.64E-03	1.00E+00	2.24E-01	1.00E+00	-2.93E+14	1.37E-05
ENSMUSG00000051457	Spn	6.E+14	5.65E-16	5.86E+14	5.43E-21	6.08E+14	9.68E-11	2.41E+14	8.67E-02	2.E-01	1.00E+00	3.E+14	7.68E-10	4.E+14	2.91E+02	-3.08E-01	1.00E+00	-1.32E-01	1.00E+00	-3.71E+14	5.12E-02
ENSMUSG00000033777	Tlr13	5.E+14	6.29E-16	4.62E+13	4.21E-45	4.81E+14	4.22E-24	2.54E+14	6.93E+09	2.E-01	1.00E+00	2.E+14	1.44E-02	2.E+14	1.69E+05	-2.82E-01	1.00E+00	-1.23E-01	1.00E+00	-2.32E+14	7.21E+05
ENSMUSG00000043008	Klhl6	5.E+14	7.56E-16	4.61E+14	2.97E-09	4.76E+14	1.10E-07	2.09E+14	5.51E-02	1.E-01	1.00E+00	2.E+14	1.50E+01	3.E+14	2.03E+03	-3.06E-01	1.00E+00	-1.92E-01	1.00E+00	-2.79E+14	1.89E-07
ENSMUSG00000054072	Iigp1	5.E+14	1.33E-15	5.63E+13	9.17E-25	5.55E+13	2.09E-25	4.83E+14	9.25E-21	-9.E-02	1.00E+00	8.E-01	8.09E-02	7.E-01	1.92E-01	4.10E-01	1.00E+00	3.03E-01	1.00E+00	-3.46E-01	6.03E-01
ENSMUSG00000060063	Alox5ap	3.E+14	1.58E-15	3.25E+14	1.27E-18	3.37E+14	2.05E-09	1.49E+14	5.14E-03	8.E-02	1.00E+00	2.E+14	2.00E+01	2.E+14	5.61E+07	1.47E-01	1.00E+00	2.19E-01	1.00E+00	-1.59E+14	1.72E+04
ENSMUSG00000039936	Pik3cd	4.E+14	3.27E-15	3.94E+14	1.17E-21	4.20E+14	2.05E-10	1.44E+13	8.45E-03	2.E-01	1.00E+00	2.E+14	2.36E-21	3.E+14	6.61E+01	-1.60E-01	1.00E+00	5.68E-02	1.00E+00	-2.63E+14	3.27E-03
ENSMUSG00000021624	Cd180	5.E+14	5.62E-15	5.13E+14	2.56E-50	5.03E+14	2.22E-22	1.85E+14	7.85E-02	-1.E-01	1.00E+00	3.E+14	3.46E-47	3.E+13	1.05E-04	-3.35E-02	1.00E+00	-1.78E-01	1.00E+00	-3.28E+14	6.29E+01
ENSMUSG00000018008	Cyth4	4.E+14	9.42E-15	3.46E+14	5.54E-15	3.77E+13	2.82E-05	1.41E+14	4.71E-03	3.E-01	1.00E+00	2.E+14	2.12E-17	2.E+14	4.40E+05	-7.96E-02	1.00E+00	1.97E-01	1.00E+00	-2.08E+14	1.20E-09
ENSMUSG00000078921	Tgtp2	5.E+14	9.42E-15	6.04E+14	7.76E-36	5.89E+14	4.72E-23	4.01E+13	6.58E+01	-2.E-01	1.00E+00	2.E+14	7.69E-07	2.E+14	1.20E+05	5.57E-01	1.00E+00	3.80E-01	1.00E+00	-1.43E+14	1.38E-03
ENSMUSG00000001281	Itgb7	5.E+14	9.57E-15	5.59E+14	4.74E-17	5.68E+14	6.40E-11	3.06E+14	4.21E+09	6.E-02	1.00E+00	3.E+14	9.97E+01	3.E+14	9.39E+06	1.71E-01	1.00E+00	2.23E-01	1.00E+00	-2.32E+13	2.54E+05
ENSMUSG00000097415	AU020206	3.E+14	1.09E-14	2.71E+14	1.72E-10	2.86E+14	1.15E-05	8.06E-01	6.78E-02	1.E-01	1.00E+00	2.E+13	5.23E-06	2.E+13	3.19E+03	-1.40E-01	1.00E+00	-2.63E-02	1.00E+00	-2.01E+14	2.00E-09
ENSMUSG00000031165	Was	4.E+14	1.66E-14	4.33E+13	2.32E-31	4.45E+13	2.30E-13	1.81E+14	2.10E-02	8.E-02	1.00E+00	2.E+14	1.52E-06	3.E+14	2.82E+05	2.66E-02	1.00E+00	9.84E-02	1.00E+00	-2.46E+14	6.63E+04
ENSMUSG00000089809	Rasgef1b	5.E+14	2.14E-14	4.54E+13	7.58E-19	4.57E+14	5.82E-08	2.15E+13	3.39E-02	-1.E-02	1.00E+00	2.E+14	4.75E-04	2.E+14	8.41E+06	-1.12E-01	1.00E+00	-1.34E-01	1.00E+00	-2.48E+14	7.48E+02
ENSMUSG00000049103	Ccr2	4.E+13	2.54E-14	4.52E+14	8.62E-20	4.50E+13	1.25E-10	1.89E+14	1.05E-03	-5.E-02	1.00E+00	3.E+13	2.35E-38	3.E+14	3.59E-03	1.32E-01	1.00E+00	7.27E-02	1.00E+00	-2.46E+14	3.85E-08
ENSMUSG00000018168	Ikzf3	5.E+14	5.30E-14	4.71E+14	2.94E-10	4.85E+14	9.75E-07	9.12E-01	6.95E-01	1.E-01	1.00E+00	4.E+13	5.78E-04	4.E+14	2.17E+02	-3.44E-01	1.00E+00	-2.49E-01	1.00E+00	-4.11E+14	2.39E-04
ENSMUSG00000076609	Igkc	8.E+14	5.79E-14	6.33E+13	1.10E+04	7.35E+14	7.19E-02	6.98E-01	9.46E-01	1.E+00	1.00E+00	6.E+14	4.66E+02	7.E+14	3.39E-05	-2.10E+14	8.32E-01	-1.14E+14	1.00E+00	-7.69E+14	8.92E-36
ENSMUSG00000013707	Tnfaip8l2	4.E+14	9.80E-14	4.37E+14	1.94E-19	4.68E+13	1.64E-10	2.25E+14	3.39E-03	3.E-01	1.00E+00	2.E+14	2.52E+00	2.E+14	6.90E+06	2.20E-01	1.00E+00	4.84E-01	1.00E+00	-1.87E+14	1.53E+05
ENSMUSG00000055385	Rnf212	-8.E+14	1.16E-13	-1.01E+14	1.10E-05	-5.90E+14	3.26E-02	-4.55E+14	9.19E+09	4.E+14	1.00E+00	-6.E+14	7.93E-02	-1.E+14	8.02E-01	-2.51E+14	1.00E+00	1.79E+14	1.00E+00	3.14E+14	2.46E-01
ENSMUSG00000000290	Itgb2	4.E+14	1.19E-13	4.00E+14	3.90E-23	4.32E+14	1.58E-08	2.19E+14	3.33E+06	3.E-01	1.00E+00	2.E+14	3.05E+01	2.E+14	1.48E+09	-1.89E-01	1.00E+00	8.71E-02	1.00E+00	-1.97E+13	4.62E+07
ENSMUSG00000030336	Cd27	5.E+14	1.45E-13	5.37E+14	8.14E-24	5.53E+14	2.08E-17	1.21E+14	7.96E-01	1.E-01	1.00E+00	4.E+14	8.58E-21	4.E+14	3.18E-10	1.91E-01	1.00E+00	3.07E-01	1.00E+00	-3.95E+14	1.28E-06
ENSMUSG00000079547	H2-DMb1	4.E+14	3.93E-13	4.67E+14	3.21E-22	4.73E+13	4.15E-13	2.65E+14	9.50E+08	3.E-02	1.00E+00	2.E+14	1.89E-01	2.E+14	5.05E+05	2.73E-01	1.00E+00	2.94E-01	1.00E+00	-1.71E+14	3.42E+06
ENSMUSG00000119584	Rn18s-rs5	-4.E+14	4.10E-13	-3.64E+14	5.55E-01	-4.20E+14	1.95E-07	-1.62E+14	3.61E+09	-6.E-01	1.00E+00	-2.E+14	9.01E-03	-3.E+14	6.91E+04	1.26E-01	1.00E+00	-4.51E-01	1.00E+00	2.18E+14	1.30E+04
ENSMUSG00000036887	C1qa	4.E+14	4.21E-13	3.95E+14	7.87E-20	4.21E+14	3.98E-12	2.04E+14	1.44E+05	2.E-01	1.00E+00	2.E+14	6.93E+00	2.E+14	5.06E+06	1.53E-01	1.00E+00	3.76E-01	1.00E+00	-1.72E+14	1.38E+07
ENSMUSG00000074785	Plxnc1	4.E+14	4.21E-13	3.91E+14	1.12E-13	3.86E+14	4.07E-07	1.85E+14	3.52E-02	-9.E-02	1.00E+00	2.E+14	6.83E+00	2.E+14	3.15E+06	-1.06E-01	1.00E+00	-2.03E-01	1.00E+00	-2.14E+14	7.91E+01
ENSMUSG00000084796	Mir142hg	6.E+14	6.32E-13	5.56E+14	1.92E-09	6.05E+13	7.87E-07	2.19E+14	3.58E-01	4.E-01	1.00E+00	3.E+14	3.54E-01	4.E+14	3.51E+04	-4.15E-02	1.00E+00	3.96E-01	1.00E+00	-3.39E+14	5.56E-03
ENSMUSG00000052336	Cx3cr1	4.E+14	6.64E-13	3.80E+13	1.38E-16	4.08E+14	8.52E-08	1.02E+14	4.20E-01	3.E-01	1.00E+00	3.E+14	5.07E-11	3.E+14	8.47E+01	-4.15E-01	1.00E+00	-1.72E-01	1.00E+00	-3.16E+14	4.95E-03
ENSMUSG00000029925	Tbxas1	4.E+14	6.72E-13	3.82E+14	3.75E-17	4.16E+14	1.59E-06	2.42E+12	4.67E+08	3.E-01	1.00E+00	1.E+14	6.62E+02	2.E+14	1.29E-04	-1.47E-01	1.00E+00	1.47E-01	1.00E+00	-1.51E+14	1.82E+07
ENSMUSG00000043931	Gimap7	7.E+14	7.93E-13	7.18E+14	1.53E-13	7.37E+14	1.74E-08	2.27E+14	7.46E-01	2.E-01	1.00E+00	5.E+14	1.09E-16	5.E+14	3.66E-05	8.06E-02	1.00E+00	2.24E-01	1.00E+00	-4.79E+14	4.13E-13
ENSMUSG00000040061	Plcb2	4.E+14	9.34E-13	4.29E+14	1.75E-28	4.43E+14	1.15E-12	1.42E+14	2.16E-01	1.E-01	1.00E+00	3.E+13	6.16E-35	3.E+14	4.03E-02	6.94E-02	1.00E+00	1.71E-01	1.00E+00	-2.76E+14	2.92E-02
ENSMUSG00000029417	Cxcl9	1.E+14	1.38E-12	1.40E+14	1.19E-15	1.41E+14	3.91E-13	1.12E+14	1.13E-01	1.E-01	1.00E+00	3.E+14	4.18E-08	3.E+14	5.91E+04	1.71E-01	1.00E+00	2.61E-01	1.00E+00	-2.52E+14	1.65E+05
ENSMUSG00000016206	H2-M3	4.E+14	1.86E-12	3.92E+14	6.59E-17	4.04E+14	4.62E-07	2.39E+14	1.78E-04	8.E-02	1.00E+00	2.E+14	1.93E+07	2.E+14	3.86E-04	7.07E-02	1.00E+00	1.42E-01	1.00E+00	-1.43E+14	2.10E+09
ENSMUSG00000038147	Cd84	5.E+14	1.94E-12	5.03E+14	1.49E-14	5.36E+14	6.13E-05	2.97E+14	2.04E+09	3.E-01	1.00E+00	2.E+14	8.37E-04	2.E+14	1.72E+09	-3.15E-01	1.00E+00	-3.63E-02	1.00E+00	-2.35E+14	3.93E+04
ENSMUSG00000025888	Casp1	4.E+14	2.27E-12	3.61E+14	1.72E-23	3.88E+14	1.63E-09	1.47E+14	2.38E-02	2.E-01	1.00E+00	2.E+14	2.06E-14	2.E+14	4.11E+04	6.76E-02	1.00E+00	2.98E-01	1.00E+00	-2.03E+13	2.50E+01
ENSMUSG00000070031	Sp140	5.E+14	2.40E-12	5.21E+14	3.84E-17	5.23E+14	2.52E-04	2.83E+14	6.21E-02	-2.E-02	1.00E+00	2.E+14	1.33E-09	2.E+14	1.03E+08	-8.19E-02	1.00E+00	-1.16E-01	1.00E+00	-2.45E+14	6.71E-03
ENSMUSG00000039146	Ifi44l	5.E+14	7.73E-12	5.35E+14	1.37E-31	4.88E+14	4.83E-18	2.43E+14	1.28E-04	-5.E-01	1.00E+00	3.E+14	1.56E-02	2.E+14	7.20E+06	5.58E-01	1.00E+00	6.02E-02	1.00E+00	-2.32E+14	3.76E+08
ENSMUSG00000026395	Ptprc	5.E+14	8.46E-12	4.84E+14	8.69E-16	4.86E+14	1.10E-05	1.61E+14	2.10E-02	-1.E-02	1.00E+00	3.E+14	5.20E-26	3.E+14	2.80E+02	-7.97E-02	1.00E+00	-1.03E-01	1.00E+00	-3.28E+14	1.71E-05
ENSMUSG00000031662	Snx20	5.E+14	8.58E-12	4.61E+14	2.98E-17	4.63E+14	1.43E-04	1.55E+14	5.35E-01	-1.E-02	1.00E+00	3.E+14	6.52E+01	3.E+14	9.48E+07	3.37E-03	1.00E+00	-2.24E-02	1.00E+00	-3.04E+14	1.39E+05
ENSMUSG00000024644	Cndp2	2.E+14	1.04E-11	2.41E+14	6.66E-12	2.54E+14	5.52E-02	8.45E-01	4.53E-02	1.E-01	1.00E+00	2.E+14	1.96E+04	2.E+14	1.82E+09	-4.29E-02	1.00E+00	5.23E-02	1.00E+00	-1.57E+14	1.55E+06
ENSMUSG00000030707	Coro1a	5.E+14	1.56E-11	4.96E+13	8.66E-12	5.06E+14	2.26E-06	1.75E+14	2.11E-02	7.E-02	1.00E+00	3.E+14	2.84E-17	3.E+14	2.01E-01	-2.48E-02	1.00E+00	3.47E-02	1.00E+00	-3.20E+14	2.15E-12
ENSMUSG00000067212	H2-T23	4.E+14	2.40E-11	4.12E+14	2.19E-15	4.10E+14	6.19E-08	2.39E+14	2.97E+07	-5.E-02	1.00E+00	2.E+14	5.99E+04	2.E+14	7.37E+09	2.03E-01	1.00E+00	1.44E-01	1.00E+00	-1.49E+14	4.91E+08
ENSMUSG00000030148	Clec4a2	4.E+14	2.40E-11	3.88E+14	1.06E-41	3.98E+14	4.24E-11	2.06E+14	4.47E-04	7.E-02	1.00E+00	2.E+14	1.45E-07	2.E+14	9.18E+08	-2.26E-02	1.00E+00	3.81E-02	1.00E+00	-1.80E+14	5.59E+08
ENSMUSG00000019850	Tnfaip3	4.E+14	2.87E-11	3.74E+14	4.13E-26	3.83E+13	2.65E-06	1.58E+14	2.13E-02	5.E-02	1.00E+00	2.E+14	4.14E-10	2.E+14	3.42E+07	2.31E-03	1.00E+00	4.52E-02	1.00E+00	-2.12E+14	7.38E+04
ENSMUSG00000024014	Pim1	3.E+14	3.36E-11	2.71E+14	3.17E-04	2.83E+14	1.78E+01	1.07E+12	1.76E-01	9.E-02	1.00E+00	2.E+14	8.50E+06	2.E+14	1.17E-04	-3.10E-01	1.00E+00	-2.27E-01	1.00E+00	-1.91E+14	7.62E+03
ENSMUSG00000027009	Itga4	4.E+14	3.46E-11	3.82E+14	1.42E-17	3.82E+14	5.47E-03	1.67E+14	2.37E-03	-3.E-02	1.00E+00	2.E+13	3.22E-09	2.E+14	4.43E+08	-2.30E-01	1.00E+00	-2.74E-01	1.00E+00	-2.35E+14	2.71E+03
ENSMUSG00000040592	Cd79b	5.E+13	3.46E-11	5.09E+14	5.94E-02	5.05E+14	1.19E+01	-3.53E+14	8.36E-02	-9.E-02	1.00E+00	9.E+14	3.10E+09	9.E+14	6.98E+09	-1.22E-01	1.00E+00	-2.22E-01	1.00E+00	-8.71E+14	3.10E+08
ENSMUSG00000027219	Slc28a2	4.E+14	3.46E-11	3.68E+14	6.68E-19	3.96E+14	6.74E-05	1.87E+14	3.90E-04	2.E-01	1.00E+00	2.E+14	2.84E-01	2.E+14	1.59E+09	6.30E-02	1.00E+00	2.99E-01	1.00E+00	-1.72E+14	3.50E+06
ENSMUSG00000036469	Marchf1	4.E+14	4.46E-11	4.28E+14	1.38E-21	4.41E+14	7.69E-06	1.90E+14	8.04E-03	9.E-02	1.00E+00	2.E+14	5.71E-03	2.E+14	3.83E+08	-5.98E-02	1.00E+00	2.22E-02	1.00E+00	-2.41E+14	4.00E+05
ENSMUSG00000056116	H2-T22	3.E+14	4.57E-11	2.92E+14	1.23E-18	2.90E+14	1.00E-08	1.41E+14	2.27E+09	-4.E-02	1.00E+00	1.E+14	5.69E+03	1.E+14	1.58E+09	2.30E-01	1.00E+00	1.74E-01	1.00E+00	-1.25E+14	6.88E+08
ENSMUSG00000018899	Irf1	3.E+13	5.61E-11	3.47E+14	6.04E-24	3.62E+14	6.07E-14	2.15E+14	3.57E+02	1.E-01	1.00E+00	1.E+14	3.91E+03	1.E+14	8.78E+08	3.15E-02	1.00E+00	1.46E-01	1.00E+00	-1.25E+14	2.38E-04
ENSMUSG00000039621	Prex1	4.E+13	5.87E-11	4.18E+14	8.91E-18	4.22E+14	4.19E-11	1.80E+14	3.59E-03	1.E-02	1.00E+00	2.E+14	3.49E-10	2.E+14	8.43E+00	-7.35E-02	1.00E+00	-7.31E-02	1.00E+00	-2.42E+13	2.02E+03
ENSMUSG00000040247	Tbc1d10c	4.E+14	6.92E-11	4.13E+14	5.84E-03	4.32E+14	7.15E-01	6.59E-01	8.99E-01	2.E-01	1.00E+00	3.E+14	1.26E+01	4.E+14	8.61E+02	-5.74E-02	1.00E+00	8.56E-02	1.00E+00	-3.49E+14	2.95E-06
ENSMUSG00000034786	Gpsm3	4.E+14	7.49E-11	3.72E+14	6.75E-11	3.83E+12	7.20E-04	1.38E+14	2.22E-01	8.E-02	1.00E+00	2.E+14	9.17E-02	2.E+14	2.28E+06	-8.01E-02	1.00E+00	-1.34E-02	1.00E+00	-2.39E+14	1.16E+00
ENSMUSG00000053318	Slamf8	6.E+13	7.77E-11	6.50E+14	8.64E-14	6.62E+14	4.23E-11	3.82E+14	4.14E-02	9.E-02	1.00E+00	3.E+14	4.91E+03	3.E+13	1.03E+07	3.54E-01	1.00E+00	4.34E-01	1.00E+00	-2.30E+14	8.27E+07
ENSMUSG00000028874	Fgr	5.E+14	7.99E-11	4.91E+14	9.58E-10	5.43E+14	1.33E-04	2.68E+14	8.99E-03	5.E-01	1.00E+00	2.E+14	6.12E+00	3.E+14	8.93E+07	-2.89E-01	1.00E+00	1.88E-01	1.00E+00	-2.48E+14	8.89E+02
ENSMUSG00000008496	Pou2f2	4.E+14	9.75E-11	3.86E+14	2.01E-22	3.98E+14	2.05E-11	1.31E+14	1.31E-01	8.E-02	1.00E+00	3.E+12	1.12E-06	3.E+13	2.79E+03	-1.79E-01	1.00E+00	-1.09E-01	1.00E+00	-2.71E+14	3.35E+03
ENSMUSG00000026117	Zap70	7.E+14	1.82E-10	6.51E+14	2.41E-11	6.70E+14	1.90E-05	2.78E+14	4.14E-01	2.E-01	1.00E+00	4.E+14	9.90E-25	4.E+14	5.80E-09	-7.39E-02	1.00E+00	8.55E-02	1.00E+00	-3.73E+14	6.45E-22
ENSMUSG00000038608	Dock10	4.E+14	3.41E-10	3.94E+14	8.53E-11	3.99E+14	1.46E-02	1.20E+14	2.03E-01	1.E-02	1.00E+00	3.E+13	3.41E-13	3.E+14	3.39E+05	-1.43E-01	1.00E+00	-1.43E-01	1.00E+00	-2.85E+14	3.00E-07
ENSMUSG00000039981	Zc3h12d	6.E+14	5.20E-10	5.72E+14	1.94E-11	5.89E+14	2.73E-01	1.66E+14	8.07E-01	1.E-01	1.00E+00	4.E+14	8.47E-24	4.E+14	6.93E-02	-4.39E-01	1.00E+00	-3.30E-01	1.00E+00	-4.50E+14	2.42E-15
ENSMUSG00000027347	Rasgrp1	4.E+14	1.50E-09	3.82E+14	5.51E-10	4.05E+14	3.30E-01	4.04E-01	9.60E-01	2.E-01	1.00E+00	3.E+14	2.71E+09	4.E+13	1.07E+08	-2.00E-01	1.00E+00	-2.14E-02	1.00E+00	-3.59E+14	1.72E+05
ENSMUSG00000024691	Fam111a	3.E+14	1.74E-09	3.32E+14	2.92E-25	3.38E+14	2.07E-05	1.19E+14	2.40E-02	3.E-02	1.00E+00	2.E+14	3.41E-13	2.E+14	4.60E+06	1.59E-01	1.00E+00	1.76E-01	1.00E+00	-1.94E+14	1.85E+04
ENSMUSG00000040747	Cd53	4.E+14	2.31E-09	4.03E+14	3.51E-16	4.18E+14	2.40E-06	1.66E+14	2.73E-03	1.E-01	1.00E+00	2.E+14	2.59E-07	2.E+14	1.13E+06	-6.69E-02	1.00E+00	3.58E-02	1.00E+00	-2.41E+13	2.47E+04
ENSMUSG00000022901	Cd86	4.E+14	2.53E-09	4.21E+13	2.21E-16	4.20E+14	2.54E-04	1.79E+14	4.41E-02	-5.E-02	1.00E+00	2.E+14	4.30E-09	2.E+14	8.79E+06	1.48E-01	1.00E+00	9.04E-02	1.00E+00	-2.23E+14	3.84E+02
ENSMUSG00000009292	Trpm2	4.E+14	4.09E-09	3.90E+13	7.70E-10	4.28E+14	1.24E-02	1.24E+14	3.72E-01	3.E-01	1.00E+00	3.E+14	1.49E-23	3.E+14	3.96E+03	-2.90E-01	1.00E+00	4.46E-02	1.00E+00	-2.92E+14	1.83E-08
ENSMUSG00000002985	Apoe	4.E+14	4.46E-09	3.54E+14	5.88E-10	3.82E+14	5.72E-07	1.59E+13	9.96E+03	3.E-01	1.00E+00	2.E+11	1.09E+06	2.E+14	1.70E+08	6.18E-03	1.00E+00	2.49E-01	1.00E+00	-1.91E+14	7.56E+06
ENSMUSG00000026822	Lcn2	4.E+14	5.69E-09	4.40E+14	7.63E-12	4.43E+14	6.52E-03	6.28E+14	2.10E-46	-5.E-03	1.00E+00	-2.E+13	6.42E+04	-2.E+14	2.69E-04	-9.65E-02	1.00E+00	-1.11E-01	1.00E+00	1.83E+13	6.42E+08
ENSMUSG00000004207	Psap	3.E+14	6.72E-09	3.29E+14	1.47E-17	3.42E+14	2.55E-10	1.40E+14	5.29E+06	1.E-01	1.00E+00	2.E+14	2.00E+01	2.E+14	3.42E+06	-1.62E-01	1.00E+00	-7.72E-02	1.00E+00	-2.02E+13	1.24E+06
ENSMUSG00000028337	Coro2a	4.E+14	7.23E-09	3.72E+14	1.86E-10	3.79E+14	6.37E-03	1.27E+14	4.00E-01	4.E-02	1.00E+00	2.E+14	4.57E-16	2.E+14	2.22E+03	-2.86E-01	1.00E+00	-2.54E-01	1.00E+00	-2.70E+13	6.76E-03
ENSMUSG00000026271	Gpr35	5.E+14	7.41E-09	5.15E+13	1.14E-15	5.10E+14	3.90E-10	2.63E+14	6.20E-02	-8.E-02	1.00E+00	2.E+14	7.40E+00	2.E+14	1.32E+06	1.37E-01	1.00E+00	5.14E-02	1.00E+00	-2.33E+14	2.00E+06
ENSMUSG00000037860	Aim2	5.E+14	7.53E-09	4.40E+14	9.90E-10	4.68E+14	1.90E-03	1.67E+14	1.41E-01	3.E-01	1.00E+00	3.E+14	1.25E-14	3.E+14	1.39E+04	-1.92E-01	1.00E+00	4.86E-02	1.00E+00	-2.88E+14	5.03E-05
ENSMUSG00000040253	Gbp7	4.E+14	8.38E-09	3.96E+13	3.19E-24	3.83E+14	2.55E-10	2.24E+14	1.96E+08	-2.E-01	1.00E+00	2.E+14	8.87E+05	2.E+14	7.93E-04	2.51E-01	1.00E+00	8.61E-02	1.00E+00	-1.44E+14	1.12E-03
ENSMUSG00000029413	Naaa	3.E+14	8.53E-09	3.63E+14	5.09E-30	3.50E+14	4.27E-09	1.05E+14	7.86E-02	-2.E-01	1.00E+00	3.E+14	1.14E-07	2.E+14	8.08E+05	2.17E-01	1.00E+00	3.90E-02	1.00E+00	-2.33E+14	5.09E+05
ENSMUSG00000016256	Ctsz	3.E+14	1.01E-08	2.58E+14	1.38E-06	2.85E+14	8.18E-03	8.98E-01	4.56E-03	2.E-01	1.00E+00	2.E+14	5.93E+04	2.E+13	2.48E+08	-1.35E-01	1.00E+00	9.98E-02	1.00E+00	-1.78E+14	1.00E+05

Table S1

ENSMUSG00000015355	Cd48	4.E+14	1.10E-08	3.89E+14	4.01E-18	4.07E+14	1.06E-07	1.61E+14	1.85E-02	1.E-01	1.00E+00	2.E+14	6.26E-08	2.E+13	2.23E+05	5.73E-02	1.00E+00	1.89E-01	1.00E+00	-2.19E+14	8.54E+04
ENSMUSG00000058470	Gm8369	4.E+13	1.26E-08	4.02E+14	1.67E-05	4.18E+14	9.74E+01	1.20E-01	9.94E-01	1.E-01	1.00E+00	4.E+14	8.13E-02	4.E+14	1.80E+05	-2.33E-01	1.00E+00	-1.25E-01	1.00E+00	-4.11E+13	9.19E-05
ENSMUSG00000049988	Lrrc25	4.E+14	1.27E-08	3.70E+14	1.50E-07	3.93E+14	9.28E-08	1.20E+13	4.00E-01	2.E-01	1.00E+00	2.E+14	1.08E-01	3.E+14	3.75E+01	-1.41E-01	1.00E+00	4.82E-02	1.00E+00	-2.60E+14	3.06E+00
ENSMUSG00000021281	Tnfaip2	4.E+14	1.33E-08	3.55E+13	6.45E-09	4.10E+14	2.56E-02	2.23E+14	3.45E+08	5.E-01	1.00E+00	1.E+14	3.81E+07	2.E+14	7.43E-04	-3.48E-02	1.00E+00	4.72E-01	1.00E+00	-1.31E+14	3.73E+06
ENSMUSG00000035202	Lars2	-3.E+14	1.80E-08	-3.32E+14	3.75E+03	-3.65E+14	1.68E+04	-1.43E+14	8.40E-04	-3.E-01	1.00E+00	-2.E+14	3.66E-02	-2.E+14	5.82E-04	1.51E-01	1.00E+00	-1.86E-01	1.00E+00	2.08E+14	8.67E-01
ENSMUSG00000020914	Top2a	4.E+14	1.97E-08	3.00E+13	2.78E-06	3.18E+14	1.32E+04	1.57E+14	1.36E-02	2.E-01	1.00E+00	1.E+14	6.18E+07	2.E+14	8.43E-03	-7.33E-01	1.00E+00	-5.93E-01	1.00E+00	-2.13E+14	5.95E+06
ENSMUSG00000096336	Igkv1-135	1.E+14	2.62E-08	9.98E+13	2.15E+01	1.22E+14	6.30E+07	4.42E+14	1.00E+00	2.E+14	1.00E+00	6.E+14	1.37E-03	8.E+14	3.88E-03	-2.76E+14	2.86E+07	-6.12E-01	1.00E+00	-8.29E+13	4.10E+08
ENSMUSG00000018920	Cxcl16	4.E+14	2.87E-08	3.80E+14	1.13E-10	3.90E+14	2.35E-04	1.98E+14	2.36E-04	8.E-02	1.00E+00	2.E+14	2.83E+00	2.E+14	3.03E+08	1.81E-01	1.00E+00	2.46E-01	1.00E+00	-1.59E+14	5.47E+03
ENSMUSG00000029204	Rhoh	6.E+14	3.47E-08	6.19E+14	1.72E-10	6.22E+14	4.36E-03	2.74E+14	2.79E-01	-1.E-02	1.00E+00	3.E+13	4.84E-08	3.E+14	9.53E+02	-1.50E-01	1.00E+00	-1.71E-01	1.00E+00	-3.59E+14	5.90E-02
ENSMUSG00000029581	Fscn1	3.E+14	6.32E-08	2.66E+14	4.97E-05	2.44E+13	7.07E+01	5.25E-01	6.83E-01	-2.E-01	1.00E+00	2.E+14	4.96E-03	2.E+14	6.77E+05	-9.18E-02	1.00E+00	-3.46E-01	1.00E+00	-2.19E+14	8.20E-07
ENSMUSG00000037337	Map4k1	4.E+14	6.86E-08	3.91E+14	1.72E-13	4.05E+14	1.21E-02	6.98E-01	7.66E-01	1.E-01	1.00E+00	3.E+14	8.28E-19	3.E+14	4.35E+04	-2.98E-01	1.00E+00	-2.12E-01	1.00E+00	-3.48E+14	8.97E-04
ENSMUSG00000029366	Dck	3.E+13	6.86E-08	3.46E+14	1.48E-20	3.16E+14	5.11E-03	1.33E+14	1.82E-01	-3.E-01	1.00E+00	2.E+14	1.78E+03	2.E+14	2.31E-04	1.59E-01	1.00E+00	-1.76E-01	1.00E+00	-1.94E+14	2.96E+08
ENSMUSG00000054717	Hmgb2	3.E+14	7.48E-08	2.64E+14	2.81E-07	2.87E+14	3.82E+03	8.90E-01	2.84E-01	2.E-01	1.00E+00	2.E+14	2.24E+04	2.E+14	1.78E-04	-4.55E-01	1.00E+00	-2.70E-01	1.00E+00	-2.17E+14	2.99E+04
ENSMUSG00000023015	Racgap1	3.E+14	7.49E-08	2.86E+14	6.29E-10	2.90E+14	8.23E+00	1.77E+14	1.61E-02	7.E-03	1.00E+00	1.E+14	2.92E-04	1.E+14	3.22E-02	-3.17E-01	1.00E+00	-3.23E-01	1.00E+00	-1.38E+14	2.10E-04
ENSMUSG00000099757	BE692007	5.E+14	8.89E-08	4.89E+14	3.90E-12	4.97E+13	3.88E-06	1.77E+14	2.27E-01	4.E-02	1.00E+00	3.E+14	2.60E-04	3.E+14	1.12E+05	2.16E-01	1.00E+00	2.44E-01	1.00E+00	-2.88E+14	4.93E+02
ENSMUSG00000024677	Ms4a6b	5.E+12	9.09E-08	5.22E+13	4.54E-14	5.30E+14	1.45E-07	2.55E+14	9.77E-04	5.E-02	1.00E+00	3.E+14	1.07E-26	3.E+14	2.23E-02	2.55E-01	1.00E+00	2.93E-01	1.00E+00	-2.38E+14	5.60E+00
ENSMUSG00000055805	Fmnl1	3.E+14	9.32E-08	3.48E+14	2.26E-19	3.60E+14	7.30E-10	6.58E-01	5.06E-01	9.E-02	1.00E+00	3.E+14	5.79E-16	3.E+14	1.12E+00	8.45E+08	1.00E+00	7.59E-02	1.00E+00	-2.79E+14	6.76E+00
ENSMUSG00000021998	Lcp1	4.E+14	1.12E-07	3.65E+14	1.54E-12	3.74E+14	2.58E-03	1.25E+14	9.74E-03	6.E-02	1.00E+00	2.E+14	1.10E-10	2.E+14	4.45E+05	-1.83E-01	1.00E+00	-1.40E-01	1.00E+00	-2.55E+14	2.48E+03
ENSMUSG00000015314	Slamf6	5.E+14	1.58E-07	5.17E+14	1.89E-04	5.40E+14	6.99E-01	-4.33E-01	9.74E-01	2.E-01	1.00E+00	6.E+13	1.73E+07	6.E+14	1.11E+02	-3.20E-01	1.00E+00	-1.39E-01	1.00E+00	-5.89E+14	8.33E+05
ENSMUSG00000052384	Nrros	3.E+14	1.77E-07	3.53E+14	1.79E-21	3.71E+14	1.89E-08	1.64E+14	4.01E-04	2.E-01	1.00E+00	2.E+14	5.12E-15	2.E+14	1.19E+06	7.84E-02	1.00E+00	2.22E-01	1.00E+00	-1.78E+14	1.51E+07
ENSMUSG00000030830	Itgal	5.E+14	1.81E-07	4.89E+14	4.14E-09	5.07E+14	1.75E-04	1.33E+14	4.26E-01	1.E-01	1.00E+00	4.E+14	1.15E-21	4.E+14	2.10E+00	-3.16E-01	1.00E+00	-1.78E-01	1.00E+00	-3.84E+14	6.27E-05
ENSMUSG00000036067	Slc2a6	4.E+14	1.84E-07	3.56E+14	6.00E-05	4.00E+14	1.73E+01	2.94E+14	4.50E+08	4.E-01	1.00E+00	6.E-01	1.48E-01	1.E+14	1.40E-01	-5.55E-02	1.00E+00	3.41E-01	1.00E+00	-6.43E-01	5.89E-02
ENSMUSG00000045322	Tlr9	8.E+14	2.26E-07	8.53E+14	2.28E-15	8.08E+14	9.66E-10	5.13E+14	1.65E-02	-5.E-01	1.00E+00	3.E+14	6.98E-25	3.E+14	9.10E-07	7.37E-02	1.00E+00	-4.22E-01	1.00E+00	-3.41E+14	6.50E+03
ENSMUSG00000042129	Rassf4	3.E+13	2.30E-07	3.46E+14	9.24E-19	3.42E+14	4.04E-07	1.75E+14	3.29E+09	-7.E-02	1.00E+00	2.E+14	4.63E-10	2.E+14	2.65E+07	2.24E-01	1.00E+00	1.45E-01	1.00E+00	-1.46E+14	1.10E+08
ENSMUSG00000021451	Sema4d	4.E+13	2.90E-07	3.59E+14	5.63E-08	3.64E+14	5.73E-01	1.06E+14	2.32E-01	2.E-02	1.00E+00	3.E+14	1.78E-09	3.E+14	2.16E+05	-2.60E-01	1.00E+00	-2.54E-01	1.00E+00	-2.76E+14	1.93E-02
ENSMUSG00000005763	Cd247	5.E+14	3.09E-07	5.41E+14	5.62E-10	5.43E+14	2.58E-03	1.71E+14	6.85E-01	3.E-03	1.00E+00	4.E+14	5.05E-12	4.E+14	1.88E+01	8.14E-02	1.00E+00	7.29E-02	1.00E+00	-3.54E+14	5.69E-07
ENSMUSG00000062545	Tlr12	8.E+14	3.78E-07	8.37E+14	3.40E-10	8.43E+14	3.07E-05	4.99E+14	7.71E-02	7.E-03	1.00E+00	3.E+14	2.21E+00	3.E+14	3.40E+07	1.50E-01	1.00E+00	1.47E-01	1.00E+00	-3.33E+13	8.01E+05
ENSMUSG00000021190	Lgmn	3.E+13	4.08E-07	3.25E+14	5.61E-12	3.42E+14	4.17E-07	1.58E+14	1.37E+09	1.E-01	1.00E+00	2.E+14	1.92E+02	2.E+14	1.03E+07	-2.06E-01	1.00E+00	-8.14E-02	1.00E+00	-1.85E+14	3.29E+07
ENSMUSG00000024669	Cd5	7.E+13	4.50E-07	6.18E+14	5.60E-06	6.59E+14	2.20E-03	1.43E+14	9.13E-01	4.E-01	1.00E+00	5.E+14	1.15E-09	5.E+14	2.76E-03	-4.15E-01	1.00E+00	-5.93E-02	1.00E+00	-5.15E+14	5.36E-08
ENSMUSG00000023947	Nfkbie	3.E+14	5.75E-07	3.23E+14	2.98E-12	3.48E+14	1.20E-07	7.14E-01	6.28E-01	2.E-01	1.00E+00	2.E+14	3.16E-02	3.E+14	1.25E+05	1.87E-02	1.00E+00	2.23E-01	1.00E+00	-2.47E+14	1.45E+04
ENSMUSG00000038213	Tapbpl	2.E+14	7.94E-07	2.45E+13	1.59E-09	2.37E+14	4.95E-04	1.15E+14	8.97E-03	-1.E-01	1.00E+00	1.E+14	4.40E+03	1.E+14	2.58E+08	9.65E-02	1.00E+00	-1.69E-02	1.00E+00	-1.17E+14	8.02E+04
ENSMUSG00000020120	Plek	5.E+14	8.13E-07	4.48E+14	2.56E-09	4.67E+14	2.82E-02	2.58E+14	1.38E-04	2.E-01	1.00E+00	2.E+14	2.00E-06	2.E+14	2.21E+09	-2.55E-01	1.00E+00	-1.11E-01	1.00E+00	-2.12E+14	3.03E+06
ENSMUSG00000051504	Siglech	4.E+14	8.36E-07	3.80E+14	1.93E-14	3.41E+14	1.63E-03	-4.11E+14	1.11E-01	-4.E-01	1.00E+00	8.E+14	8.37E-09	7.E+13	9.75E-04	2.18E-01	1.00E+00	-2.11E-01	1.00E+00	-7.67E+14	6.35E-06
ENSMUSG00000030165	Klrd1	4.E+13	1.02E-06	4.29E+14	2.89E-06	4.25E+14	2.49E-03	1.54E+14	2.55E-01	-6.E-02	1.00E+00	3.E+14	7.04E+03	3.E+14	8.53E+06	3.44E-01	1.00E+00	2.72E-01	1.00E+00	-2.38E+14	6.40E+03
ENSMUSG00000052085	Dock8	4.E+14	1.14E-06	3.68E+14	8.32E-15	3.67E+14	8.54E-06	1.27E+14	4.76E-02	-4.E-02	1.00E+00	2.E+13	1.32E-03	2.E+14	3.05E+06	-2.37E-01	1.00E+00	-2.90E-01	1.00E+00	-2.62E+14	1.81E+05
ENSMUSG00000047810	Ccdc88b	4.E+14	1.44E-06	4.23E+14	8.90E-05	4.60E+14	7.08E-02	2.21E+14	1.60E-02	3.E-01	1.00E+00	2.E+14	1.43E-06	2.E+14	2.70E+06	-1.69E-01	1.00E+00	1.53E-01	1.00E+00	-2.15E+13	1.23E-05
ENSMUSG00000043004	Gng2	4.E+14	1.47E-06	3.53E+14	1.46E-17	3.55E+13	2.88E-06	1.15E+14	2.72E-01	-2.E-02	1.00E+00	2.E+13	7.57E-09	2.E+14	3.13E+04	-9.65E-02	1.00E+00	-1.26E-01	1.00E+00	-2.44E+14	1.72E+04
ENSMUSG00000030748	Il4ra	3.E+14	1.51E-06	2.96E+14	4.42E-07	3.07E+14	2.48E+00	5.66E-01	6.85E-01	8.E-02	1.00E+00	2.E+14	1.21E-07	2.E+14	5.56E+04	-7.08E-02	1.00E+00	-4.65E-03	1.00E+00	-2.43E+14	8.29E-03
ENSMUSG00000027322	Siglec1	4.E+14	1.74E-06	4.29E+14	2.62E-27	4.31E+14	9.41E-14	2.71E+14	2.60E+04	-4.E-03	1.00E+00	2.E+14	7.46E+01	2.E+14	8.40E+09	2.74E-02	1.00E+00	1.14E-02	1.00E+00	-1.52E+13	2.73E-03
ENSMUSG00000029915	Clec5a	6.E+14	2.03E-06	5.56E+12	5.82E-10	5.99E+14	9.23E-04	3.99E+14	6.53E-03	4.E-01	1.00E+00	2.E+14	4.88E+04	2.E+13	4.80E+09	-1.23E-01	1.00E+00	2.47E-01	1.00E+00	-1.70E+14	2.20E+08
ENSMUSG00000102037	Bcl2a1a	7.E+14	2.03E-06	6.76E+14	4.05E-06	7.03E+14	4.71E-02	2.94E+14	6.56E-01	2.E-01	1.00E+00	4.E+14	7.17E-01	4.E+14	1.35E+06	-9.41E-02	1.00E+00	1.10E-01	1.00E+00	-4.00E+14	2.40E+02
ENSMUSG00000031004	Mki67	4.E+14	3.75E-06	2.94E+14	1.31E-09	3.55E+14	1.18E+04	2.18E+14	1.24E-04	6.E-01	1.00E+00	7.E-01	9.18E-02	1.E+14	1.14E-01	-1.22E+14	2.83E-01	-6.57E-01	1.00E+00	-1.95E+14	1.37E-03
ENSMUSG00000021298	Gpr132	5.E+14	3.76E-06	4.75E+13	1.22E-06	5.04E+14	1.02E-01	2.34E+14	2.76E-01	3.E-01	1.00E+00	2.E+14	2.04E+00	3.E+13	3.27E+07	-3.43E-01	1.00E+00	-8.07E-02	1.00E+00	-2.69E+14	4.57E+02
ENSMUSG00000002603	Tgfb1	3.E+14	4.70E-06	3.25E+14	1.24E-14	3.35E+14	4.45E-02	1.25E+14	1.05E-02	6.E-02	1.00E+00	2.E+13	1.01E+02	2.E+13	1.51E+09	-7.36E-02	1.00E+00	-2.71E-02	1.00E+00	-2.04E+14	2.38E+07
ENSMUSG00000070034	Sp110	4.E+14	4.72E-06	4.08E+14	5.17E-12	4.20E+13	1.39E-06	1.80E+13	1.40E-02	9.E-02	1.00E+00	2.E+14	1.72E-07	2.E+14	2.88E+03	1.06E-01	1.00E+00	1.82E-01	1.00E+00	-2.14E+13	1.27E+05
ENSMUSG00000035697	Arhgap45	4.E+14	4.83E-06	3.86E+13	5.50E-06	4.00E+14	4.18E-01	1.26E+13	6.80E-02	1.E-01	1.00E+00	3.E+14	1.36E-08	3.E+14	5.90E+04	-1.70E-02	1.00E+00	6.85E-02	1.00E+00	-2.58E+14	7.39E-06
ENSMUSG00000005087	Cd44	3.E+13	5.38E-06	2.97E+14	1.95E-06	3.06E+14	2.33E+00	1.11E+14	6.86E-02	6.E-02	1.00E+00	2.E+12	2.23E-01	2.E+14	6.22E+07	-1.29E-01	1.00E+00	-7.85E-02	1.00E+00	-1.96E+14	4.82E+03
ENSMUSG00000030724	Cd19	5.E+14	6.84E-06	5.24E+14	1.95E+01	5.21E+13	1.26E+04	-9.62E-01	8.02E-01	-8.E-02	1.00E+00	6.E+14	1.44E+06	6.E+13	1.63E+01	9.49E-03	1.00E+00	-7.65E-02	1.00E+00	-6.16E+14	2.24E-20
ENSMUSG00000034422	Parp14	3.E+14	7.00E-06	3.85E+14	4.09E-22	3.56E+14	2.93E-16	2.02E+14	1.12E+07	-3.E-01	1.00E+00	2.E+14	4.37E+02	1.E+14	2.26E+08	4.20E-01	1.00E+00	1.02E-01	1.00E+00	-1.37E+14	1.36E-03
ENSMUSG00000021262	Evl	3.E+13	7.44E-06	3.16E+14	5.20E-11	3.24E+14	3.50E-02	1.09E+14	1.36E-01	5.E-02	1.00E+00	2.E+14	2.43E-06	2.E+14	3.18E+06	1.71E-02	1.00E+00	5.61E-02	1.00E+00	-2.02E+14	1.48E+03
ENSMUSG00000024030	Abcg1	3.E+13	7.50E-06	3.42E+14	4.97E-17	3.28E+14	1.34E-08	7.93E-01	3.68E-01	-2.E-01	1.00E+00	3.E+14	1.17E-04	2.E+14	1.92E+04	1.21E-01	1.00E+00	-5.41E-02	1.00E+00	-2.47E+14	1.12E+05
ENSMUSG00000000486	Septin1	4.E+14	7.63E-06	3.49E+14	4.87E-04	3.76E+14	5.61E-03	3.23E-01	9.39E-01	2.E-01	1.00E+00	3.E+14	9.50E-06	3.E+14	2.16E+00	-4.15E-01	1.00E+00	-1.99E-01	1.00E+00	-3.56E+14	2.05E-03
ENSMUSG00000004707	Ly9	5.E+14	8.13E-06	4.96E+14	2.97E-09	5.10E+14	1.94E-03	2.28E+14	2.45E-02	1.E-01	1.00E+00	3.E+14	2.79E-08	3.E+14	2.67E+05	-1.73E-01	1.00E+00	-7.99E-02	1.00E+00	-2.83E+14	1.89E+04
ENSMUSG00000096422	Igkv12-44	1.E+14	8.91E-06	1.08E+14	1.37E+06	1.05E+14	2.00E+09	3.65E+14	1.00E+00	-3.E-01	1.00E+00	7.E+14	2.47E-04	7.E+14	8.16E-03	-1.46E+14	1.00E+00	-1.79E+14	1.00E+00	-8.59E+14	4.80E+07
ENSMUSG00000024965	Fermt3	3.E+14	9.30E-06	3.36E+14	7.46E-10	3.62E+14	3.23E-02	1.09E+14	1.39E-01	2.E-01	1.00E+00	2.E+14	1.23E-16	2.E+14	1.09E+05	-1.29E-01	1.00E+00	9.06E-02	1.00E+00	-2.36E+14	6.35E+00
ENSMUSG00000026417	Pigr	4.E+14	9.74E-06	3.97E+14	1.10E-31	3.49E+13	2.60E+07	2.48E+14	8.87E+04	-5.E-01	1.00E+00	1.E+14	2.41E+07	1.E+00	3.40E-01	1.95E-01	1.00E+00	-3.13E-01	1.00E+00	-1.27E+14	1.55E-02

Figure S1

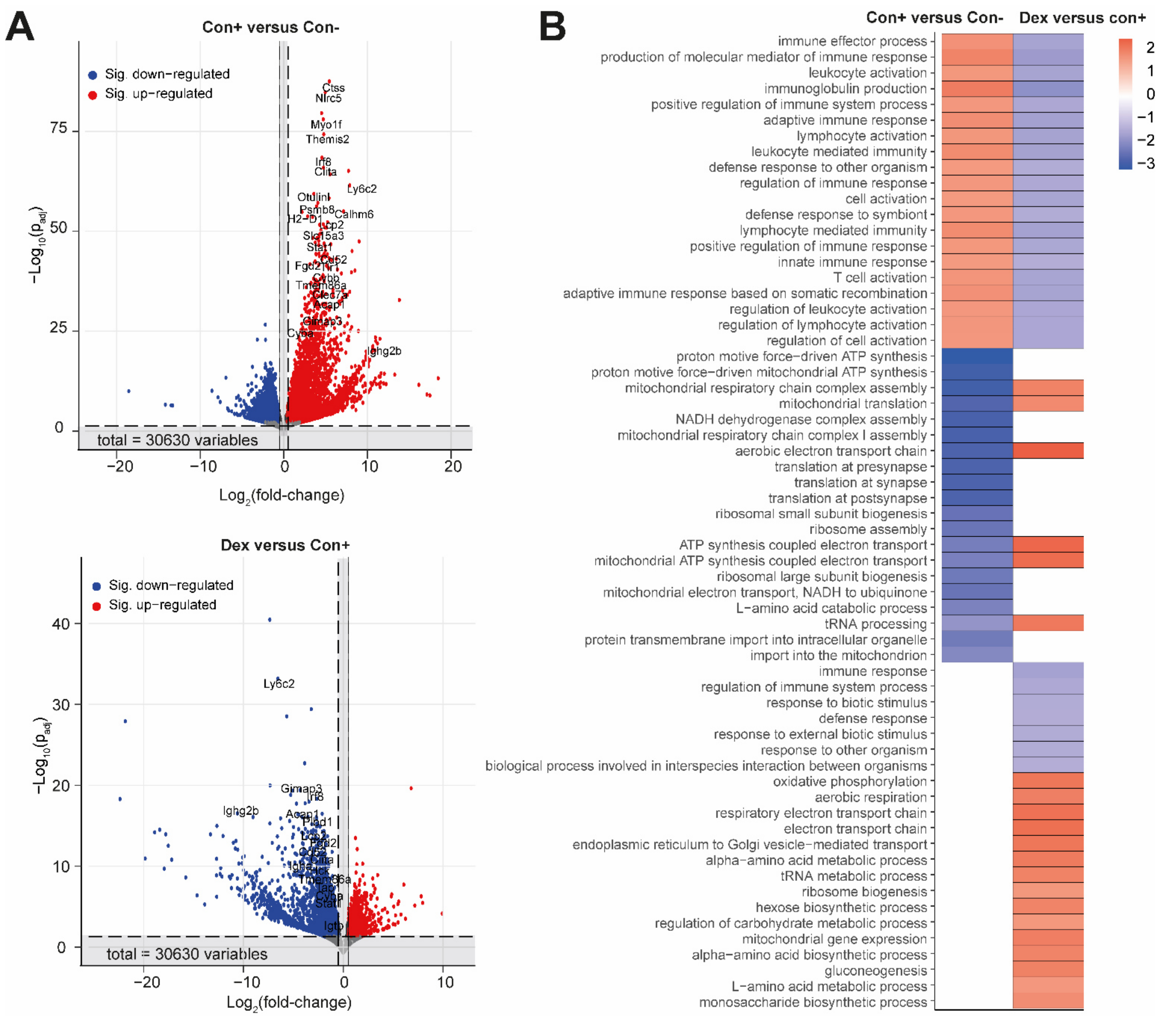
A
Con+ versus Con-
Sig. down−regulated
Sig. up−regulated
total = 30630 variables
−Log10(padj)
Log2(fold-change)
Dex versus Con+
Sig. down−regulated
Sig. up−regulated
total = 30630 variables
−Log10(padj)
Log2(fold-change)
B
Con+ versus Con-
Dex versus con+
immune effector process
production of molecular mediator of immune response
leukocyte activation
immunoglobulin production
positive regulation of immune system process
adaptive immune response
lymphocyte activation
leukocyte mediated immunity
defense response to other organism
regulation of immune response
cell activation
defense response to symbiont
lymphocyte mediated immunity
positive regulation of immune response
innate immune response
T cell activation
adaptive immune response based on somatic recombination
regulation of leukocyte activation
regulation of lymphocyte activation
regulation of cell activation
proton motive force−driven ATP synthesis
proton motive force−driven mitochondrial ATP synthesis
mitochondrial respiratory chain complex assembly
mitochondrial translation
NADH dehydrogenase complex assembly
mitochondrial respiratory chain complex I assembly
aerobic electron transport chain
translation at presynapse
translation at synapse
translation at postsynapse
ribosomal small subunit biogenesis
ribosome assembly
ATP synthesis coupled electron transport
mitochondrial ATP synthesis coupled electron transport
ribosomal large subunit biogenesis
mitochondrial electron transport, NADH to ubiquinone
L−amino acid catabolic process
tRNA processing
protein transmembrane import into intracellular organelle
import into the mitochondrion
immune response
regulation of immune system process
response to biotic stimulus
defense response
response to external biotic stimulus
response to other organism
biological process involved in interspecies interaction between organisms
oxidative phosphorylation
aerobic respiration
respiratory electron transport chain
electron transport chain
endoplasmic reticulum to Golgi vesicle−mediated transport
alpha−amino acid metabolic process
tRNA metabolic process
ribosome biogenesis
hexose biosynthetic process
regulation of carbohydrate metabolic process
mitochondrial gene expression
alpha−amino acid biosynthetic process
gluconeogenesis
L−amino acid metabolic process
monosaccharide biosynthetic process
